# A Comparative Study on UHPLC-HRMS Profiles and Biological Activities of *Inula sarana* Different Extracts and Its Beta-Cyclodextrin Complex: Effective Insights for Novel Applications

**DOI:** 10.3390/antiox12101842

**Published:** 2023-10-10

**Authors:** Gokhan Zengin, Nilofar, Evren Yildiztugay, Abdelhakim Bouyahya, Halit Cavusoglu, Reneta Gevrenova, Dimitrina Zheleva-Dimitrova

**Affiliations:** 1 Physiology and Biochemistry Laboratory, Department of Biology, Science Faculty, Selcuk University, Konya 42130, Turkey; nilofar.nilofar@unich.it; 2 Department of Pharmacy, Botanic Garden “Giardino dei Semplici”, Università degli Studi “Gabriele d’Annunzio”, via dei Vestini 31, 66100 Chieti, Italy; 3 Department of Biotechnology, Science Faculty, Selcuk University, Konya 42130, Turkey; eytugay@selcuk.edu.tr; 4 Laboratory of Human Pathologies Biology, Faculty of Sciences, Mohammed V University in Rabat, Rabat 10106, Morocco; a.bouyahya@um5r.ac.ma; 5 Department of Physics, Science Faculty, Selcuk University, Konya 42130, Turkey; hcavusoglu@selcuk.edu.tr; 6 Department of Pharmacognosy, Faculty of Pharmacy, Medical University of Sofia, 1000 Sofia, Bulgaria; rgevrenova@pharmfac.mu-sofia.bg

**Keywords:** *Inula sarana*, β-cyclodextrin, chemical characterization, SEM, enzyme inhibitory

## Abstract

Within this particular framework, the extracts obtained from *Inula sarana* using a variety of solvents, included n-hexane, ethyl acetate, dichloromethane (DCM), 70% ethanol, ethanol, and water. The extracts obtained from n-hexane, ethyl acetate, and DCM were then subjected to a specific method for their incorporation into β-cyclodextrin (β-CD). The establishment of complex formation was validated through the utilization of scanning electron microscopy (SEM) and Fourier Transform Infrared Spectroscopy (FTIR). The identification of phytochemical components was executed using UHPLC-HRMS. Furthermore, the total phenolic and flavonoid content was evaluated using the Folin–Ciocalteu assay and the AlCl_3_ method. Subsequently, the determination of antioxidant capacity was conducted utilizing DPPH, ABTS, CUPRAC, Frap, PBD, and MCA assays. The enzyme inhibitory activities of the samples (extracts and β-CD complexes) were also examined by AChE, BChE, tyrosinase, α-glucosidase, and α-amylase. The findings indicated that water and 70% ethanol extracts contained the highest phenolic content. One hundred and fourteen bioactive compounds were identified by UHPLC-HRMS analysis. This study unveiled a substantial array of flavonoids, phenolic acid-hexosides and caffeoylhexaric acids within *I. sarana*, marking their initial identification in this context. Among the various extracts tested, the 70% ethanol extract stood out due to its high flavonoid content (jaceosidin, cirsiliol, and eupatilin) and hydroxybenzoic and hydroxycinnamic acid hexosides. This extract also displayed notably enhanced antioxidant activity, with ABTS, CUPRAC, and FRAP test values of 106.50 mg TE/g dry extract, 224.31 mg TE/g dry extract, and 110.40 mg TE/g, respectively. However, the antioxidant values of the complex extracts with β-CD were generally lower than those of the pure extracts, an observation warranting significant consideration. In terms of enzyme inhibition activity, the ethanol and 70% ethanol extracts exhibited higher inhibitory effects on AChE, tyrosinase, and α-glucosidase. Conversely, n-hexane displayed stronger inhibitory activity against BChE. The ethyl acetate extract demonstrated elevated amylase inhibitory activity. However, the antioxidant values of the complex extracts with β-CD were generally lower than those of the pure extracts, a noteworthy observation, while water and extracts from the *I. sarana* complex with β-CD exhibited minimal or negatable inhibitory activity against specific enzymes.

## 1. Introduction

The Asteraceae family, comprising the largest group of flowering plants, encompasses over 24,000 to 30,000 species and 1600 to 1700 genera [1]. Within this family are found essential botanical resources, including medicinal oil-yielding plants, horticultural resources, and economically significant species. Noteworthy phytochemical constituents like polyphenols, diterpenoids, and flavonoids are prevalent among the constituents of this botanical family [2]. Therefore, species of this family typically stand out for their notable antioxidant, anti-inflammatory, antiparasitic, and anticancer properties [3,4]. The genus *Inula* L. encompasses approximately 25 species, with nine endemic to Turkey, is predominantly distributed in Asia, Africa, Europe, and is notably abundant in the Mediterranean region [5,6,7].

The limited solubility of many drugs in water represents one of the most significant challenges preventing the advancement of various pharmaceuticals. A notable observation reveals that approximately 40% of newly developed drugs display poor water solubility, which prevents their efficient clinical application [8]. This challenge has led to the exploration of various precise drug encapsulation techniques. One particular strategic possibility involves the encapsulation of such drugs using various methods. This approach has the potential to increase dissolution rates, increase membrane permeability, and, ultimately, improve the bioavailability of nutraceuticals with low solubility. Generally, cyclodextrins (CDs) are low-cost starch derivatives that have been manufactured and enzymatically modified. These nontoxic starch derivatives have minimal absorption in the upper gastrointestinal tract and are completely metabolized by the gut microbes [9]. The unique hollow molecular structure of β-cyclodextrin (β-CD) allows it to form inclusion complexes with a diverse range of organic compounds. These compounds partially or completely accommodated within its relatively hydrophobic cavity, eliminating a limited number of high-energy water molecules [9,10]. The pharmaceutical industry frequently utilizes cyclodextrins due to their ability to form inclusion complexes with various molecules, serving as a means to enhance the water solubility and consequently, the bioavailability of lipophilic drugs. This strategy, though, is less effective for hydrophilic or moderately polar drugs, leading to the exploration of cyclodextrin derivatives. However, these challenges can be effectively addressed through chemical modifications. β-CD, categorized as a polysaccharide, has some limitations, including insufficient water solubility and possible toxicities when ingested intravenously [11]. These challenges, however, can be effectively addressed through chemical modifications. The approach of synthesizing nanosponges based on cyclodextrins appears to be a viable technique to bolster and prolong the stability of drugs.

Curcumin’s clinical applicability is limited due to challenges like low bioavailability, minimal solubility in aqueous settings, and susceptibility to chemical degradation at physiological pH levels; however, studies have demonstrated that its solubility can be enhanced by up to 2.34 times through complexation with β-CD [12]. In a study recently published, a remarkable enhancement of 206-fold in curcumin solubility was documented, demonstrating the sustained release of curcumin from the resultant inclusion complexes [13]. Recently, a number of studies have reported the successful encapsulation of extracts and essential oils derived from the Asteraceae family. This encapsulation process has been designed to enhance the bioavailability of bioactive compounds contained within these extracts and oils. The ultimate goal is to significantly boost their efficacy in various applications. This development represents a notable advancement in the field of natural product encapsulation and opens up new possibilities for harnessing the therapeutic potential of these bioactive substances [14,15,16]. Nevertheless, scientific exploration into the characteristics of *Inula sarana* remains limited. Hence, due to the unexplored nature of *I. sarana*, we undertook a comprehensive study of this particular species.

Beyond their pharmaceutical applications, cyclodextrins (CDs) provide a number of benefits with broader implications. Their ability to extend the shelf life of food products has sparked interest, demonstrating their significance beyond the realm of medicine. Moreover, CDs have the ability to mask or eliminate unpleasant tastes and odours, addressing concerns about the flavour of certain goods [17]. The process of encapsulating plant extract within β-CD at the molecular level holds numerous potentials for several benefits. As study claimed that olive leaf encapsulation in β-CD demonstrates the potential for various enhancements, including increased solubility in aqueous solutions, improved compatibility within oil/water systems, enhanced protection against oxidative degradation during storage, and the possibility of improving its bioavailability [10,18].

In this context, the study aimed to determine the chemical composition (by using UHPLC-HRMS) of *I. sarana* extracts and their biological activities Additionally, we investigated the changes in chemical profiles and biological properties in β-CD incorporation of its ethyl acetate, n-hexane, and DCM. This aspect can provide significant information for preparing formulations using *I. sarana* for further pharmaceutical, nutraceutical, and cosmeceutical applications.

## 2. Materials and Methods

### 2.1. Chemicals

Acetonitrile, formic acid (for LC–MS) and methanol (analytical grade) were provided from Merck (Merck, Sofia, Bulgaria). The reference standards, using for UHPLC-HRMS protocatechuic, *p*-coumaric, *m*-coumaric, *o*-coumaric, vanillic, gentisic and salicylic acids, luteolin 7-*O*-glucoside, kaempferol-3-*O*-rutinoside, kaempferol 3-*O*-glucoside, isorhamnetin 3-*O*-glucoside, nepetin 7-*O*-glucoside, hyperoside, apigenin, quercetin, kaempferol, hispidulin, chrysoeriol, isorhamnetin, jseocidine, and luteolin were obtained from Extrasynthese (Genay, France). Chlorogenic, neochlorogenic, caffeic, 3,4-dicaffeoylquinic, 3,5-dicaffeylquinic and 1,5-dicaffeoylquinic acids, as well as dehydrocostus lactone, isoalantolactone, alantolactone, and partenolide, were supplied from Phytolab (Vesten-Bergsgreuth, Germany).

### 2.2. Plant Material

In the summer of 2021, the aerial parts of *I. sarana* were gathered in Konya (Turkey: Seydisehir-Antalya road, Alacabel location, 1640 m) by the botanist Evren Yildiztugay. After collecting the plants, one specimen was deposited at the Selcuk University herbarium (EY-3212). The plant materials underwent a thorough washing process with tap and distilled water to eliminate any soil and contaminants prior to extraction. The aerial parts were then air-dried for 10 days in shade at room temperature and powdered.

### 2.3. Sample Preparation

The n-hexane, ethyl acetate, DCM, 100% ethanol, and 70% ethanol extracts were obtained through the maceration method, wherein 10 g of plant material was mixed with 200 mL of each solvent and steeped for 24 h at room temperature. We used Whatman 1 filter paper to filter the mixtures and a rotary-evaporator to remove the solvents. To make the water extract (100%), we boiled 200 milliliters of water and infused 10 g of plant material for 15 min. After filtration, we lyophilized the extract for 48 h. Subsequently, all extracts were stored at 4 °C until analysis. The extraction yields were 3.32% (for n-hexane), 4.33% (for DCM), 5.03% (for ethyl acetate), 5.90% (for ethanol), 17.03% (for 70% ethanol) and 6.63% (for water).

### 2.4. Total Phenolic and Flavonoids Content

Total phenolic (Folin–Ciocalteu assay) and flavonoids (AlCl_3_ assay) contents of the extracts/complexes by colorimetric method using the describe method for phenolic by Acquaviva et al. [19] and for flavonoids by Llorent-Martínez et al. [20]. Results were expressed as gallic acid equivalents (mg GAE/g dry extract or complex) and rutin equivalents (mg RE/g dry extract or complex) for respective assays.

### 2.5. UHPLC-HRMS Analysis

The UHPLC-HRMS analyses were performed as previously described [21]. Q Exactive Plus mass spectrometer with a heated electrospray ionization (HESI-II) probe (ThermoFisher Scientific, Inc., Waltham, MA, USA), operated in negative and positive ion modes (*m/z* range from 100 to 1500 at a resolution of 70,000) was used for the LC-MS analyses. The tune parameters were as follows: 2.5 kV spray voltage, 38 AU sheath gas flow rate, 12 AU auxiliary gas flow rate, 320 °C capillary temperature, 320 °C probe heater temperature, and level 50 S-lens RF. Acquisition was developed at the full-scan MS and Data Dependent-MS2 modes (DD-MS2). Other parameters for Full MS mode were as follows: maximum injection time (IT) 100 ms, number of scan ranges 1, and automatic gain control (AGC) target 3 × 10^6^. For the DD-MS2 mode, the instrument parameters were maximum IT 50ms, microscans 1, resolution 17,500, AGC target 1 × 10^5^, MSX count 1, Top5, and isolation window 2.0 *m/z*, and the stepped normalized collision energy was 10, 20, and 60 eV. The separation was performed on a C18 column Kromasil EternityXT (1.8 µm, 2.1 × 100 mm) (Bohus, Ale, Sweden) at 40 °C. The mobile phase contained 0.1% formic acid in water (A) and 0.1% formic acid in acetonitrile (B). The run time was 33 min, and the flow rate was 0.3 mL/min. The gradient elution program was as follows: 0–1 min, 5% B; 1–20 min, 5–30% B; 20–25 min, 30–50% B; 25–30 min, 50–70% B; 30–33 min, 70–95%; and 33–34 min, 95–5% B. Equilibration time was 4 min. The injection volume was set to 1 µL, and the flow rate was 300 µL/min. Data acquisition was performed using Xcalibur 4.2 software (Thermo Scientific). MZmine 2.53 software was applied to the UHPLC–HRMS raw files of the *I. sarana* extract for further analysis.

### 2.6. Preparation and Characterization of β-CD Complexes

The inclusions for n-hexane, ethyl acetate and dicholoromethane (DCM) extracts were prepared in a 1:1 ratio. Fifty mg of extracts were dissolved in 50 mL of methanol and these extracts were carefully filtered. Then, almost 50 mg β-CD (in 50 mL of water) was added and the mixture was stirred at room temperature for 24 h. After that, the mixtures were filtered, and the methanol was removed using a rotary evaporator. The final mixtures were lyophilized at −40 °C to remove all water and the dry samples were stored in the dark condition at 4 °C. The obtained inclusions were named hexane/β-CD, ethyl acetate/β-CD and DCM/β-CD.

The samples were directly placed on aluminum stabs; then, the shape and surface characteristics were observed by micrographs of the samples taken by scanning electron microscopy (SEM) (Zeiss EVO LS10; Oberkochen, Germany) using the gold sputter technique. The zone magnification for the images were kept around 20,000×. Observations were performed under 1 and 15 kV. Fourier transform infrared spectrum (FTIR) was recorded on a Vertex 70 Bruker FTIR spectrometer (Bruker, Leipzig, Germany) with attenuated total reflectance (ATR) module. All spectra were captured in the 400 and 4000 cm^−1^ spectral region at a scan rate of 180 scans and a spectral resolution of 4 cm^−1^. The FTIR spectrum was used in the transmittance mode.

### 2.7. In Vitro Antioxidant Assays

We evaluated the antioxidant potential of the extracts through the execution of six complementary in vitro spectrophotometric tests, as described in reference [22]. These tests included examinations of the ABTS and DPPH assays, which probe the extracts’ ability to neutralize free radicals. The extract’s ability to reduce (FRAP and CUPRAC assays) and its metal chelating capacity (MCA) were evaluated. Total antioxidant ability was measured by the phosphomolybdenum (PBD) assay. We utilized the Trolox standard for assessing all assays except MCA, for which we compared the results in terms of equivalent EDTA per gram of extract.

### 2.8. Enzyme Inhibitory Activity

The inhibitory effects of extracts and inclusions were investigated against different enzymes acetylcholinesterase (AChE), butyrylcholinesterase (BChE), tyrosinase, α-amylase and α-glucosidase. The enzyme inhibitory assays were performed as reported by Zengin [23]. The inhibition of AChE and BChE was quantified as milligrams of galanthamine equivalents (GALAE) per gram of extract, while tyrosinase inhibition was measured as milligrams of kojic acid equivalents (KAE) per gram of extract. In addition, α-amylase and α-glucosidase inhibition were assessed in terms of millimoles of acarbose equivalents (ACAE) per gram of extract.

### 2.9. Statistical Analysis

Statistical analysis was performed using Xl Stat (Version 16). All analyses were conducted in triplicates (*n* = 3) and presented as mean values with their standard deviation (mean value ± std). Differences between samples were examined using one-way analysis of variance (ANOVA) and Tukey’s post hoc test with significance level set at *p* < 0.05. Veen diagrams were designed using an online platform (http://bioinformatics.psb.ugent.be/webtools/Venn/; accessed on 20 August 2023). Circle heat map was also obtained from an online platform (https://www.chiplot.online/; accessed on 20 August 2023). Graph Pad Prism (version 9.2) was used for Pearson correlation analysis.

## 3. Results and Discussion

### 3.1. Characterization of β-CD Complexes

As because polar solvent extracts are typically not encapsulated by β-CD due to the strong hydrophilic interactions between polar compounds and water, which limit their inclusion within the hydrophobic cavity of β-CD [24]. So, water, 70% ethanol, and water extracts was not encapsulated with β-CD. The n-hexane, ethyl acetate and DCM extracts were incorporated into β-CD, and were characterized.

#### 3.1.1. SEM Analysis

**Figure 1:**
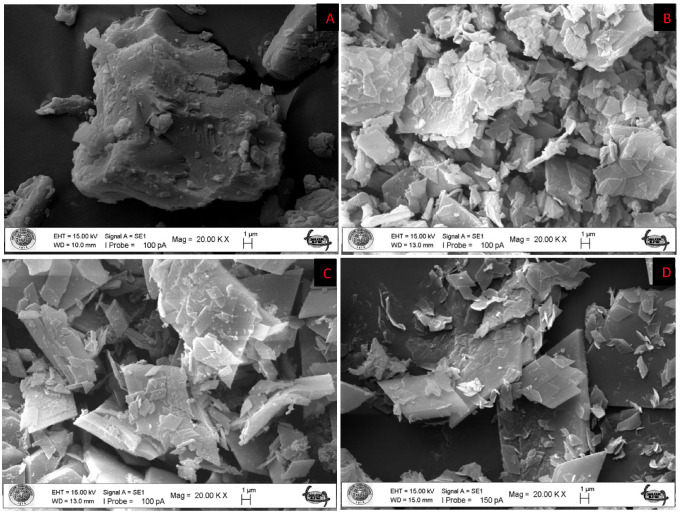
SEM images of pure β-cyclodextrin (**A**); Ethyl acetate/β-cyclodextrin (**B**); Hexane/β-cyclodextrin (**C**); DCM/β-cyclodextrin (**D**) inclusion complex.

When it comes to scrutinizing the microscopic morphology and structure of solids, especially crystalline materials, scanning electron microscopy (SEM) serves as an indispensable tool [25]. A concentrated electron beam is used to scan a sample’s surface in order to create images of it. By interacting with the atoms in the sample, the electrons create signals that provide information about the sample’s surface topography and chemical composition. The morphologies of pure β-CD, ethyl acetate/β-CD, hexane/ β-CD and DCM/β-CD were characterized by SEM and the images are shown in Figure 1. The structural differences observed among the related complexes imply that additional crystalline phases may have emerged following complexation. In general, the β-CD particles (Figure 1) displayed irregular and parallelogram shapes under various inclusion complexes.

#### 3.1.2. Fourier Transform Infrared Spectroscopic Analysis

**Figure 2:**
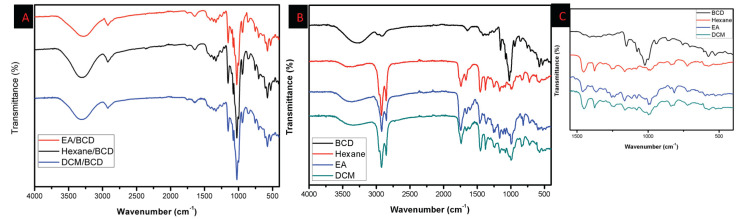
FT–IR spectra of Ethyl acetate/β–cyclodextrin, Hexane/β–cyclodextrin, DCM/β–cyclodextrin inclusion complex (**A**), FT–IR spectra of pure β–cyclodextrin and each extract without the presence of β–cyclodextrin (**B**), with enlarged form between 1550 cm^−1^ and 400 cm^−1^ (**C**).

The infrared spectrum’s position and intensity correspond to the features of molecular structures. The production of inclusion complex and the interaction of ethyl acetate, n-hexane, DCM, and β-CD can be inferred from changes in FTIR spectra. The FTIR spectra of ethyl acetate/β-CD, n-hexane/β-CD, and DCM/β-CD inclusion complex were shown in Figure 2A. In addition, FTIR spectra of pure β-cyclodextrin and each extract without the presence of β-cyclodextrin with enlarged form between 1550 cm^−1^ and 400 cm^−1^ are shown in Figure 2B The typical absorption frequency of β-CDs spans the full 400–3800 cm^−1^ range. Organic small molecules can only constitute a maximum of 25% (*w/w*) of the inclusion complex [26]. Their distinctive peaks are obscured and hard to distinguish by the absorption peaks of β-CDs. Tensile vibration absorption occurs about 1700 cm^−1^ if the guest molecule has a group like –COOH, –COOR, or C=O. The stretching (–OH) vibration of hydroxyl groups causes the broad peak around 3380 cm^−1^. According to Figure 2, the stretching (C-H) vibration is responsible for the peaks at 2970 and 2900 cm^−1^ in the FTIR spectrum of all samples, while the (C–H) deformation vibration is responsible for the peak at 1390 cm^−1^. The FTIR spectrum exhibits two peaks as a result of the stretching (C–O) vibration. The stretching (C–O) vibration of the co-carbon carboxyl group causes one peak to occur at 1210 cm^−1^, and the stretching (C–O) vibration of the –O–CH_2_CH_3_ group causes another comparably strong peak to appear at 1040 cm^−1^ [27].

The composition of different extracts of *I. sarana* and its complex with β-CD has been studied in terms of total phenolic content and total flavonoid content. The results have been categorized among various solvents and different extraction conditions.

### 3.2. Total Phenolic and Flavonoid Content

Among the various extracts obtained, the highest total phenolic content was found in the extract obtained from water, with a value of 55.55 mg GAE/g dry extract (Table 1). On the other hand, the lowest total phenolic content was observed in the n-hexane extract, which had a value of 12.45 mg GAE/g dry extract. In the middle range, the ethanol extract had a total phenolic content of 38.42 mg GAE/g dry extract. In terms of the total flavonoid content, the highest value was recorded for the extract obtained from ethyl acetate, with 22.60 mg RE/g dry extract. Conversely, the lowest total flavonoid content was found in the water extract, which had a value of 9.11 mg RE/g dry extract. In the middle range, the DCM extract exhibited a total flavonoid content of 16.57 mg RE/g dry extract. When considering the extracts obtained from the complex of *I. sarana* with β-CD, the highest total phenolic content was observed in the dichloromethane/β-CD (DCM/β-CD), with a value of 4.54 mg GAE/g dry complex, followed by the ethyl acetate/β-CD had a total phenolic content of 4.17 mg GAE/g dry complex. The lowest total phenolic content in this group was found in the n-hexane/β-CD, which had a value of 3.00 mg GAE/g dry complex. In terms of total flavonoid content in the β-CD complex extracts, the highest value was recorded for the ethyl acetate/β-CD, with 3.82 mg RE/g dry complex. However, n-hexane/β-CD had lowest total flavonoid content 1.96 ± 0.26 mg RE/g dry complex.

These results highlight variations in the levels of total phenolic and flavonoid compounds, depending on the solvents and extraction conditions as well as the effect of complexation with β-CD on these levels.

The results of this study on the extracts of *I. sarana* as well as its complex with β-CD provide significant insights into the phenolic and flavonoid composition of this medicinal plant and its complex. Recognized for their antioxidative features, these compounds may have positive implications for human health.

The variations observed in the total phenolic and flavonoid contents based on the extraction solvents indicate that different solvents have varying capabilities to extract these compounds from the plant. For instance, extracts obtained from water and 70% ethanol appear to have the highest contents of phenolic and flavonoid compounds. This suggests that these solvents are more effective in extracting these compounds from the plant [28,29,30]. In our study, the polar solvents, namely ethanol, 70% ethanol, and water, were generally rich in terms of total phenolic content. This can be attributed to the enhanced solvation of antioxidant compounds in the extracts, which is caused by the interactions (especially hydrogen bonds) between the polar sites of the antioxidant molecules and these solvents. In addition, both lipophilic and hydrophilic phenolics can be extracted by the solvents and thus can result in high values in the spectrophotometric assays. This fact was also confirmed by UHPLC-HRMS analysis, which showed more components in the polar solvents when compared to nonpolar solvents.

The results of the *I. sarana*/β-CD complex extracts generally showed lower total phenolic and flavonoid contents compared to the classical extracts. Understanding these observations is important for product formulation involving extracts, where a reduction in phenolic and flavonoid compounds might impact their potential effectiveness. Therefore, further research and experimentation are imperative to identify the precise reasons behind this decrease. It is important to acknowledge that the explanation may vary depending on the specific plant extract, extract amount, and experimental conditions. Studies prove the concentration of β-CD and the ratio at which it forms complexes with the relevant extracts playing a significant role. The not optimized ratio may lead to reduced antioxidant activity [31]. Additional studies are necessary to gain deeper insights and validate these findings. This unexpected decrease in phenolic and flavonoid content when using β-CD challenges conventional assumptions, encouraging researchers to explore novel mechanisms and applications. The deviation from the typical enhancement effect of β-CD on phenolic and flavonoid content may complicate the formulation of products reliant on these extracts, potentially affecting their intended effectiveness.

### 3.3. UHPLC-HRMS Analysis

**Figure 3:**
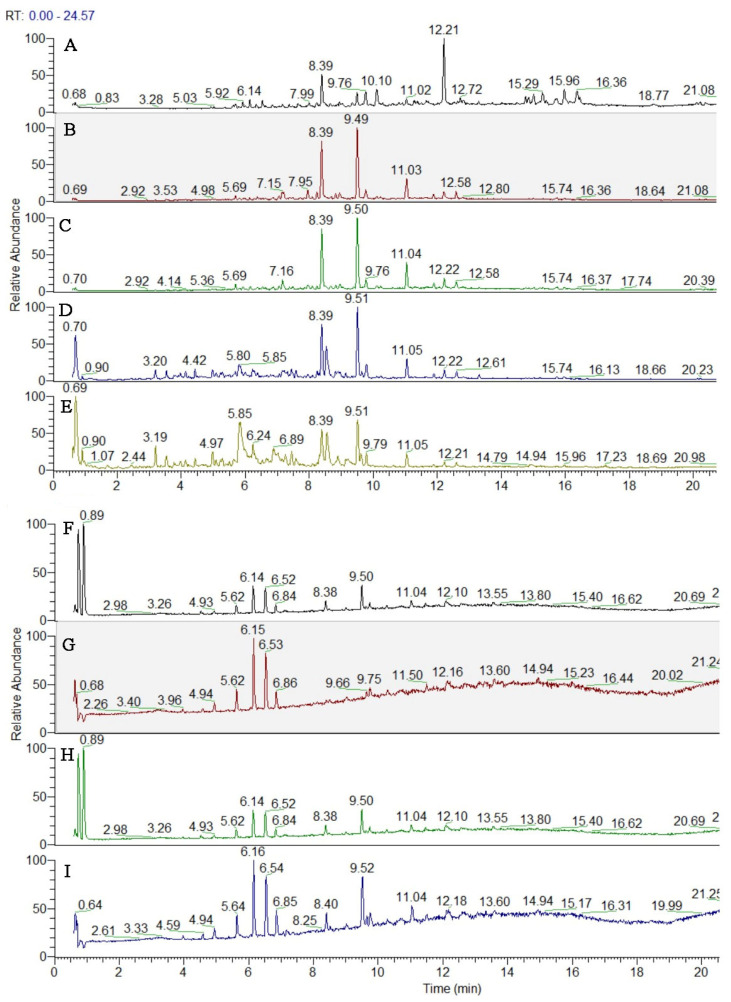
TIC of studied *Inula sarana* extracts in negative ion mode; (**A**) n-hexane extract; (**B**) ethyl acetate extract; (**C**) DCM extract; (**D**) ethanol extract; (**E**) 70% ethanol extract; (**F**) water extract; (**G**) n-hexane/β-CD; (**H**) ethyl acetate/β-CD; (**I**) DCM/β-CD.

The compound identification/annotation approach was based on the fragmentation rules and indicative ions for different types of compounds, reference standards and literature data [21]. The main points in the peak’s annotation and dereplication are accurate masses in Full MS, MS/MS fragmentation, relative abundance of deprotonated/protonated molecules and fragment ions, elemental composition, correspondence to the simulated monoisotopic profiles, and comparison with the retention times, MS/MS spectra and chromatographic behavior of reference standards. The total ion chromatograms (TIC) are given in Figure 3.

**Figure 4:**
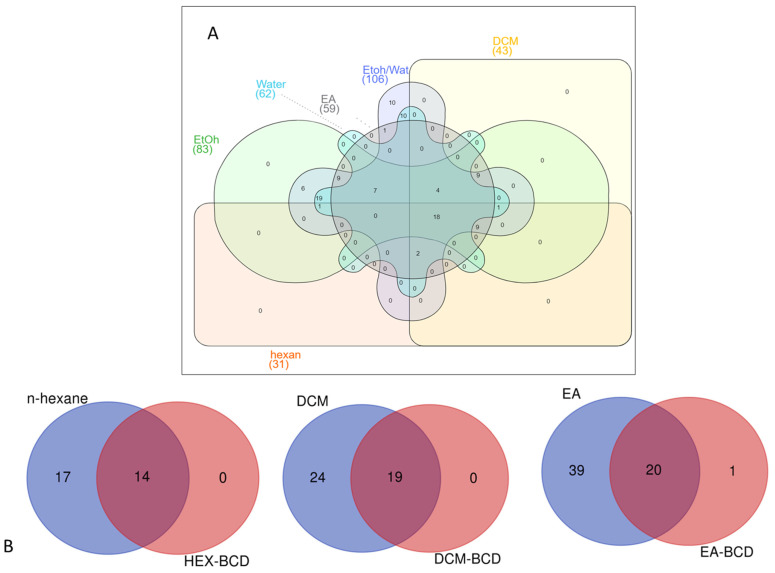
Venn diagrams showing number of compounds identified from the tested extracts (**A**). Comparison the identified compounds in the extracts and theirs complexes (**B**). EA: Ethyl acetate; DCM: Dichloromethane; EtOh: Ethanol; EtOh/Water: 70% Ethanol; BCD: β-cyclodextrin.

To compare the number of identified compounds in the tested extracts, we constructed Venn diagrams, and the results are presented in Figure 4. As can be seen from Figure 4A, most compounds were identified in the 70% ethanol extract with 106 compounds, followed by ethanol (83) and water (62). The n-hexane extract contained the lowest number of chemicals (31). In this sense, the polar solvents can be suggested for further application with *I. sarana*. Furthermore, we compared the number of identified compounds in the extracts and their complex. From Figure 4B, it can be seen that almost 45–50% of the identified compounds were protected by β-CD complexation.

#### 3.3.1. Hydroxybenzoic and Hydroxycinnamic Acids and Their Derivatives

Several glycosides of hydroxybenzoic and hydroxycinnamic acids, including hexosides 1–4, 6, 8–11 and 17 were tentatively identified (Tables S1 and S2, Figure S1). A variety of the phenylpropanoid glycosides were also evidenced. Thus, compounds 31 and 33 demonstrated the same deprotonated molecules [M–H]^−^ at *m/z* 521.130 (calc. for C_24_H_25_O_13_). The occurrence of caffeoyl and syringyl residues was deduced from the transitions 521.130→323.077 [M–H–C_9_H_10_O_5_]^−^ and 323.077→161.023 [M–H–C_6_H_10_O_5_]^−^, supported by the indicative fragment ions at *m/z* 179.034 [caffeic acid–H]^−^ and 197.045 [syringic acid–H]^−^. Additionally, the syringic acid was evidenced by the fragment ions at *m/z* 182.021 [syringic acid–H–CH_3_]^−^, 166.998 [syringic acid–H–2CH_3_]^−^ and 123.008 [syringic acid–H–2CH_3_–CO_2_]^−^. Accordingly, 31 and 33 were assigned to syringic acid-(caffeoyl)-hexosides. In the same manner, the transitions 441.140→323.077 [M–H–C_5_H_10_O_3_]^−^ and 323.077→161.023 [M–H–C_6_H_10_O_5_]^−^ indicated both hydroxyisovaleryl and caffeoyl moieties in 35 (caffeic acid-(hydroxyisovaleryl(-hexoside). In addition, coumaric acid at *m/z* 163.060, gentisic acid at *m/z* 153.018, hydroxybenzoic acid at *m/z* 137.023 and vanillic acid at *m/z* 167.034 were dedicated in 32, 34, 36/43 and 37, respectively. Thus, they were ascribed as hydroxyisopropanoic acid-(coumaroyl)-hexoside, gentisic acid-(caffeoyl)-hexoside, caffeic acid-(hydroxybenzoyl)-hexoside and vanillic acid-(caffeoyl)-hexoside, respectively.

Six hydroxybenzoic acids (5, 12, 15, 16, 25 and 42) and 3 hydroxycinnamic acids (22, 24, and 30) together with quinic acid (20) and two caffeoylgluconic ester isomers (13 and 18) were identified in the extracts on the basis of comparison with the reference standards, respectively (Table S1, Figure S1).

Although hydroxybenzoic and hydroxycinnamic acids were found in their free form, herein, a great number of their hexosides and phenylpropanoid glycosides were evidenced in *I. sarana* for the first time. Quinic acid (21) was the main compound in this group of compounds in the studied extract, together with 10 and 15 (Figure S1).

#### 3.3.2. Acylquinic Acids (AQAs)

Herein, 5 *mono*AQA and 7 *di*AQA and 1 *tri*AQA acids were annotated/dereplicated in the *I. sarana* extracts (Tables S1 and S2, Figure S2). The AQAs recognition was based on the fragment ions and their relative abundances corresponding to each subclass AQAs. The base peaks at *m/z* 191.055 was indicative for a substitution at C-5 of quinic acid skeleton. Thus, 21, 26 and 28 were assigned to 5-caffeoyl-, 5-*p*-coumaroyl and 5-feruloylquinic acid, respectively.

*di*AQA consisted of the following subclasses: dicaffeoylquinic acids (*di*CQA) (38–41), *p*-coumaroyl-caffeoylquinic acids (*p*-CoCQA) (44, 45) and hydroxydihydrocaffeoyl-caffeoylquinic acids (HC-CQA) (29).

Compounds 40 and 44 gave diagnostic ions at *m/z* 353.088, indicating a loss of caffeoyl (40) and *p*-coumaroyl (44) moiety. Moreover, both compounds gave base peaks at *m/z* 191.055 together with the prominent ion at *m/z* 179.034 [caffeic acid–H]^−^ (40) and 163.039 (44), as was observed in 3-*mono*AQA (Table S1). Thus, 40 and 44 were ascribed as 3, 5-*di*CQA and 3C-5-*p*-CoQA, respectively.

Vicinal *di*CQA 3, 4-*di*CQA (38), 4,5-*di*CQA (41) and 4*p*-Co-5CQA (45) were evidenced by the diagnostic “dehydrated” quinic acid ion at *m/z* 173.045 (Table S1).

Peak 46 yielded a precursor ion at *m/z* 677.152 (calc. for C_34_H_29_O_15_), along with the transitions at *m*/*z* 677.152→515.120→353.088→191.055, resulting from the losses of three caffeoyl residues. 4,5-*di*substituted quinic acid skeleton was deduced from the fragment ions at *m*/*z* 191.055 (48%), 179. 034 (68%), 173.045 (92%) and 135.044 (81%). Thus, 46 was assigned to 3,4,5-*tri*caffeoylquinic acid.

#### 3.3.3. Caffeoylhexaric Acids (CHAs)

Overall, 4 *mono*CHA, 5 *di*CHA, 3 *tri*CHA and 2 *tetra*CHA were annotated together with their esters with aliphatic acids and hexosides. The starting points in the CHAs recognition were the diagnostic fragment ions resulting from the subsequent losses of one (47–50), two (51–55), three (57–59) and four (56 and 60) caffeoyl residues (Table S1, Figure S3). Accordingly, the base peak at *m*/*z* 209.030 (C_6_H_9_O_8_) [hexaric acid (HA)–H]^−^ was observed along with a number of fragment ions at *m*/*z* 191.019 [HA–H–H_2_O]^−^, 173.009 [HA–H–2H_2_O]^−^, 147.029 [HA–H–H_2_O–CO_2_]^−^, 129.018 [HA–H–2H_2_O–CO_2_]^−^, 111.007 [HA–H–3H_2_O–CO_2_]^−^, 85.028 [HA–H–2H_2_O–2CO_2_]^−^ (47) (Table S1) [21,32]. Compounds 62 and 63 shared the same [M–H]*^−^* at *m*/*z* 617.153 (calc. for C_29_H_29_O_15_). They afforded the indicative fragment ions at *m*/*z* 293.088 [M–H–2caffeoyl]^−^ and 191.019 [M–H–2caffeoyl–C_5_H_10_O_2_]^−^ resulting from the losses of caffeoyl residues and a subsequent loss of 102.069 Da (calc. for C_5_H_10_O_2_) or 2-methylbutiric acid/isovaleric acid (62). Caffeoyl moiety was deduced from the fragment ions at *m*/*z* 179.034 [caffeic acid (CA)–H]^−^*,* [(CA–H)–H_2_O]^−^ at *m/z* 161.023 and 135.044 [(CA–H)–CO_2_]^−^. Accordingly, 62 and 63 were ascribed as isomeric 2-methylbutanyl/isovaleryl*-*dicaffeoylhexaric acids. In the same manner, 66 and 67 were annotated as 2-methylbutanyl/isovaleryl-tricaffeoylhexaric acid. Similarly, the assignment of 65 ([M–H]^−^ at *m*/*z* 765.169, C_37_H_33_O_18_) was suggested by the transition 279.072→191.019, resulting from the loss of 88.054 Da (calc. for C_4_H_8_O_2_) or isobutyric acid. Concerning compounds 61 and 64, they afforded the indicative fragment ions at *m*/*z* 279.072 [M–H–3caffeoyl-Hex]^−^ and 293.088, respectively, before the loss of the corresponding aliphatic acid. Accordingly, 64 was assigned to isobutanyl-tricaffeoylhexaric acid-hexoside (61) and 2-methylbutanyl/isovaleryl-tricaffeoylhexaric acid-hexoside (64).

#### 3.3.4. Flavonoids

Overall, 6 flavone-, 10-flavonol- and 1 flavanone-glycosides were found. The dereplication/annotation of flavonoids was based on the indicative ions and their relative abundances in MS/MS spectra, corresponding to the respective flavonoid’s subclass reported elsewhere [21].

The sugar chain of 68–70, 72, 73, 78 and 80 was consistent with rutinoside (308.112 Da, C_12_H_20_O_9_) (Tables S1 and S2, Figure S4). Exemplifying this consistency, in (−)ESI-MS/MS the precursor ions at *m/z* 623.163 (80) generated deprotonated molecule of the aglycone (Y_0_^−^) at *m/z* 315.051. The aglycone was apparent by the prominent fragment at *m/z* 300.028 [Y_0_–H–^•^CH_3_]^−^, supported by a series of neutral and radical losses and RDA ions at *m/z* 271.025 [Y_0_–H–CH_3_–CHO^•^]^−^, 243.030 [Y_0_-H–CH_3_–CHO^•^–CO]^−^, 227.034 [Y_0_–H–CH_3_–CHO^•^–CO_2_]^−^ and 215.035 [Y_0_–H–CH_3_–CHO^•^–2CO]^−^ (Table S1). Methoxylation at a ring A of the 6-hydroxyluteolin was evident by the RDA ions at *m/z* 165.990 (^1,3^A^−^–CH_3_), 133.028 (^1,3^B^−^), as was seen in patuletin. Accordingly, 80 was ascribed as nepetin *O*-rutinoside. Quercetin (68, 69, 71, 74 and 77) and luteolin (72 and 75) were proved by the base peaks and RDA ions at 151.002 (^1,3^A^−^) and 107.012 (^0,4^A^−^), 178.998 (^1,2^A^−^), 121.029 (^1,2^B^−^) (quercetin), and 133.028 (^1,3^B^−^) (luteolin). Compounds 68 and 69 revealed the same [M–H]^−^ at *m/z* 609.147. The aglycone quercetin was evidenced in both flavonoids: 68 afforded a base peak (Y_0_^−^) at 301.036, while 69 gave (Y_0_^−^) at *m/z* 301.035 together with an abundant fragment ion of the radical aglycone [Y_0_–H]^•−^ at *m/z* 300.028 (68.6%), suggesting the 3-*O*-glycosidic bond [33]. Accordingly, 68 was identified as rutin, additionally confirmed with the standard reference, while 69 was annotated as quercetin 7-*O*-rutinoside. Isoquercitrin (71), hyperoside (74), luteolin 7-*O*-glucoside (75), kaempferol 3-*O*-glucoside (81) and isorhamnetin 3-*O*-glucoside (82) were clearly identified by comparison with reference standards.

The approach for recognition of 6-methoxylated flavonoids was delineated elsewhere [21]. Among them, 7 methoxylated derivatives of quercetagetin (85, 88, 90, 94, 101, 102 and 105), four 6-hydroxyluteolin derivatives (89, 91, 100 and 104), and two scutelarein derivatives (96 and 103) were described.

As an example, isomers 94, 101 and 102 ([M–H]^−^ at *m/z* 359.077, C_18_H_15_O_8_) demonstrated the fragmentation pathway of 6-methoxylated quercetagetin derivatives (Table S1, Figure S5). In (−)ESI-MS/MS 94, 101, and 102 yielded radical losses at *m/z* 344.054 [M–H–^•^CH_3_]^−^, 329.031 [M–H–2^•^CH_3_]^−^ and 314.009 [M–H–3^•^CH_3_]^−^. A series of neutral losses were also recorded at *m/z* 301.036 [M–H–2^•^CH_3_–CO]^−^, 286.012 [M–H–3^•^CH_3_–CO]^−^, 258.071 [M–H–3^•^CH_3_–2CO]^−^ and 230.022 [M–H–3^•^CH_3_–3CO]^−^. In addition, a series of RDA ions were observed at *m/z* 165.990 (^1,3^A^−^–^•^CH_3_), 136.987 (^1,3^A^−^–CO–CH_4_) and 109.999 (^1,3^A^−^–CO–CHO–CH_3_) indicating methoxylation in the A-ring. A dimethoxylated RDA ion ^1,3^B was discernable by prominent fragment ions at *m/z* 163.040 (^1,3^B^−^–CH_2_) and 148.016 (^1,3^B^−^–^•^CH_3_–CH_2_) (Table S1). Thus, 94, 101 and 102 were ascribed as quercetagetin-3,6,3′(4′)-trimethyl ether isomers.

#### 3.3.5. Sesquiterpene Lactones and Derivatives

The dereplication of sesquiterpene lactones and derivatives was performed by UHPLC-ESI/HRMS of *I. sarana* extracts in positive ion mode as more relevant for the analysis of this class of specialized metabolites [34]. Based on accurate masse in Full MS, MS/MS fragmentation pathways, relative abundance of precursor and fragment ions, elemental composition, and comparison with reference standards and literature data, six sesquiterpene lactones and three sesquiterpenes were identified or tentatively annotated in *I. sarana* extracts. Previous investigation revealed MS/MS fragmentation patterns of sesquiterpene lactones with characteristic ions corresponding to the loss of H_2_O (−18 Da), CO (−28 Da), 2xH_2_O (−36 Da), and CO_2_ (−44 Da), CH_3_COOH (−60 Da), as well as concomitant loss of H_2_O + CO (−46 Da), 2xH_2_O + CO (−64 Da), and H_2_O + CO_2_ (−62 Da) [35].

Two peaks shared the same 108 and 113 protonated ion [M+H]^+^ at *m/z* 233.153. The abundant fragments at *m/z* 215.143 (55.6% for 108) and (60.4% for 113) [M+H–H_2_O]^+^ are in agreement with a presence of OH group. The base peak at *m/z* 187.148 [M+H-H_2_O–CO]^+^, together with diagnostic fragments at *m/z* 161.132 [M+H–CO–CO_2_]^+^, *m/z* 117.070 [C_9_H_8_C_9_H_9_]^+^ and *m/z* 105.070 [C_8_H_8_C_9_H_9_]^+^, as well as comparison to the reference standards allowed the identification of 108 and 113 as isoalantolactone and alantolactone, respectively. Similarly, 107 and 110 were related to hydroxyalantolactone and dehydroalantolactone, respectively. In addition, based on a comparison with reference standards, 106 was related to dehydrocostus lactone, while 109 to partenolide.

### 3.4. Antioxidant Properties

The thorough analysis of the antioxidant activity of *I. sarana* extracts and its complex with β-CD revealed intriguing diversity in results. The DPPH, ABTS, CUPRAC, FRAP, PBD, and MCA tests provided essential insights into the antioxidant capacities of each sample, uncovering significant trends. The ethanol extract displayed high values in the ABTS (73.61 mg TE/g dry extract), CUPRAC (159.00 mg TE/g dry extract), and FRAP (74.18 mg TE/g dry extract) tests, reflecting substantial antioxidant activity. Interestingly, the 70% ethanol extract exhibited even more impressive antioxidant activity, with values of 106.5 mg TE/g dry extract in the ABTS test, 224.31 mg TE/g dry extract in the CUPRAC test, and 110.40 mg TE/g dry extract in the FRAP test (Table 2). The equations, range and R2 values of standard compounds in the biological activity assays are presented in Table S3.

However, the extracts form the complex with β-CD generally showed lower antioxidant values compared to the pure relevant extracts. For instance, the n-hexane extract exhibited DPPH and ABTS scavenging ability as 17.95 mg TE/g dry complex and 12.02 mg TE/g dry complex, respectively; however, the incorporation of the extracts in β-CD negatively affects its ability (DPPH: 15.17 mg TE/g; ABTS: no active). A similar case was also noted for ethyl acetate and DCM. These observations suggest that complexation with β-CD might influence the availability and reactivity of antioxidant compounds.

First and foremost, the observation of significant differences in antioxidant capacities among the various extracts underscores the critical influence of solvent choice in the extraction process. Ethanol-based extracts particularly excelled in multiple tests, illustrating ethanol’s efficacy in solubilizing antioxidant compounds. The outstanding outcomes obtained with the 70% ethanol extract underscore the potential in extracting active compounds, which could be attributed to the increased polarity properties [36,37,38].

Regarding the extracts from the complex with β-CD, the trend of displaying reduced antioxidant values deserves particular attention. This observation suggests that the formation of the complex might have consequences on the release, stability, or accessibility of antioxidant compounds, potentially due to their encapsulation within the β-CD cavity [39,40]. This intricate interaction between the complex and active compounds could hold significant implications for the design and utilization of formulations based on these extracts. Previous studies have indicated that in vitro approaches using antioxidants demonstrate superior efficacy compared to drug complexes with CDs [41]. This difference can be attributed to the potential formation of stable phenoxy radicals through intermolecular hydrogen bonding between the hydroxyl groups of CDs and hydroxyl-containing drugs, particularly for those with hydroxyl groups [42,43]. CDs are also known to enhance drug stability by reducing oxidation [44]. Therefore, the complexation of CDs may not be advantageous for in vitro models involving extracts rich in polyphenols with hydroxyl groups. Additionally, it has been reported that complex antioxidant abilities may not be detectable using certain in vitro test systems, such as DPPH, FRAP, and CUPRAC, as these methods measure distinct properties of the complex [44,45].

Nonetheless, a study carried out by a group of researchers, as indicated by Escobar-Avello et al., yielded results that demonstrated minimal distinctions between the effects of pure extracts and encapsulated extracts [46]. For instance, when investigating encapsulated β-CD, it was revealed that their antioxidant capability measured at 5300 µmol TE/g [46] was quite comparable to the reading of 4612 µmol TE/g obtained from the pure extract [47]. This observation underscores that the encapsulation process utilizing β-CD seemed to have a negligible impact on enhancing the antioxidant potential of the compound.

On the contrary, numerous authors propose the notion that the antioxidative potential of phenolic compounds experiences enhancement through their encapsulation within β-CD [48]. This encapsulation process within cyclodextrins is believed to bolster the antioxidant capacity of phenolic compounds, according to several scholarly perspectives.

**Figure 5:**
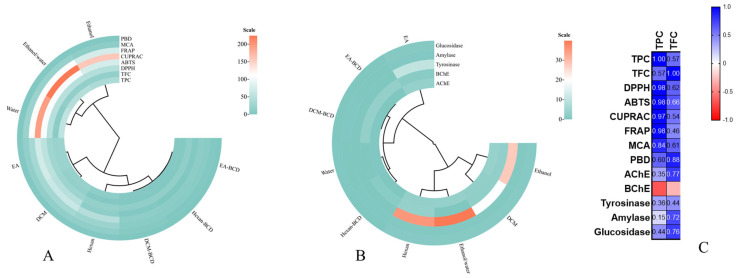
Circle heat maps for the results from antioxidant (**A**) and enzyme inhibitory assays (**B**) and Pearson correlation values between total bioactive compounds and biological activities (**C**) (TPC: Total phenolic content; TFC: Total flavonoid content; ABTS: 2,2′-azino-bis(3-ethylbenzothiazoline-6-sulphonic acid; DPPH: 1,1-diphenyl-2-picrylhydrazyl; CUPRAC: Cupric reducing antioxidant capacity; FRAP: Ferric reducing antioxidant power; AChE: acetylcholinesterase; BChE: butyrylcholinesterase; MCA: Metal chelating assay; PBD: Phosphomolybdenum).

Furthermore, the variation in results across different antioxidant tests reflects the diversity of mechanisms by which the compounds present in the extracts can scavenge free radicals and other reactive species. The DPPH, ABTS, CUPRAC, FRAP, PBD, and MCA tests utilize distinct mechanisms to assess antioxidant activity, which can explain why certain active substances exhibit superior performance in a specific test but not in others. To gain more insights into the relationship between bioactive compounds and antioxidant abilities, we designed circle and Pearson’s correlation heat maps. Based on the circle heat map, the extracts/complex were divided into different groups. Polar extracts, namely ethanol, 70% ethanol and water, were divided into the same group. Others (ethyl acetate, DCM, and n-hexane) were placed in a different group. In addition, the β-CD complexes were very close to each other (Figure 5A). Based on Pearson’s correlation values, the total phenolic content was highly correlated with radical scavenging, reducing and metal chelating abilities (R > 0.8).

The observed antioxidant ability of the extracts can be attributed to the presence of certain compounds. Chlorogenic and other caffeoylquinic acids, which are common in the Asteraceae family, are main players in the antioxidant mechanisms of chlorogenic acid due to their hydroxyl groups [49]. Rutin, which was detected in all extracts, also plays an essential role in the antioxidant properties of the extracts due to its 3′ OH group [50]. In addition, 4-hydroxybenzoic and 3-hydroxybenzoic acids, which were detected in all samples, are significant antioxidants with hydroxyl groups [51]. Sesquiterpenes are also important groups in the chemical composition of the tested samples and can contribute to the observed antioxidant properties. For example, partenolide has been reported as a protective shield against oxidative stress in moderate dosages [52]. Therefore, the antioxidant ability of the extracts can be attributed to the presence of these compounds.

In summary, these results shed light on the complexity of the antioxidant activity of *I. sarana* extracts and their complex with β-CD. They underscore the importance of understanding the interactions between active compounds, solvents, and complex forms to optimize the antioxidant properties of the extracts. This study provides crucial insights to guide future research aimed at exploring underlying mechanisms and harnessing these extracts for applications beneficial to human health.

### 3.5. Enzyme Inhibition Effects

#### 3.5.1. α-Amylase and α-Glucosidase

Diabetes, also referred to as diabetes mellitus, stands as one of the most widespread epidemic diseases globally [53]. It is characterized by the inhibition of enzyme activities responsible for the conversion of dietary starch into glucose, namely α-amylase and α-glucosidase [54]. Nature has consistently proven to be a generous reservoir of valuable compounds known for their potential health benefits. Among these, plants emerge as a substantial source of secondary metabolites, notably phenolic and flavonoid compounds, with recognized inhibitory properties against α-amylase and α-glucosidase activities [55].

The investigation into the inhibitory activity of *I. sarana* and its complex with β-CD on the enzymes amylase and glucosidase has unveiled intriguing insights into their potential as regulators of carbohydrate digestion. The results reveal significant variations in inhibition values among the different extracts and the complex. Extract obtained from ethyl acetate exhibited the highest inhibition values for amylase, averaging at 0.49 mmol ACAE/g dry extract. This suggests that these extracts might contain compounds capable of modulating amylase activity, which is involved in breaking down starch into simple sugars. The n-hexane and DCM extracts also displayed notable inhibitory activity against amylase, with values of 0.47 and 0.44 ACAE/g dry extract, respectively. Conversely, water extract exhibited lower inhibition values, indicating less pronounced amylase inhibitory activity (Table 3).

Regarding glucosidase, 70% ethanol extract showcased the highest inhibitory activity, with average values of 0.93 mmol ACAE/g dry extract. This might suggest that this extract contain compounds capable of modulating the digestion and absorption of simple carbohydrates. DCM extract also exhibited high inhibition values for glucosidase, with values of 0.90 mmol ACAE/g. Conversely, the water extract displayed weaker inhibitory activity, indicating a limited impact on glucosidase.

The inhibitory activity observed in the extracts of the complex with β-CD might suggest a potential modulation of digestive enzymes through complex formation, although further in-depth research is needed to understand the underlying mechanisms.

The results from the evaluation of the inhibitory activity of *I. sarana* and its complex with β-CD on the enzymes amylase and glucosidase provide significant insights into their potential in regulating carbohydrate digestion, with implications for glycemic management and metabolic health.

The observed variations in inhibition values among the different extracts and the complex underscore the importance of selecting the appropriate extraction solvent for the final composition of the extracts and their inhibitory activity [36,56]. Seventy percent ethanol extract exhibited the highest inhibition values for both amylase and glucosidase, suggesting that these extracts might contain specific bioactive compounds capable of modulating the activity of these digestive enzymes [57,58,59]. The ethanol extract also displayed significant inhibitory activity, confirming the positive impact of ethanol as an extraction solvent.

The observation of lower inhibitory activities in water extract suggests that the bioactive compounds responsible for the inhibitory activity might be more soluble in organic solvents. This raises questions about the specific compounds present in the organic extracts that are responsible for this inhibitory activity.

The formation of the complex with β-CD exhibited some inhibitory activity, although the results are less conclusive due to the presence of “na” values for certain extracts. However, this could suggest that complex formation may potentially influence the interactions between the extracted compounds and the target enzymes, thereby modulating their activity. This phenomenon can be elucidated by considering the involvement of hydroxyl groups in phenolic and flavonoid compounds in the formation of β-CD complexes. The depletion of free hydroxyl groups in these compounds may lead to a reduction in enzyme inhibition.

#### 3.5.2. Cholinesterase Inhibitory Activity

Alzheimer’s Disease (AD) is a globally recognized neurodegenerative condition that has sparked widespread concern, currently affecting over 35 million individuals across the world [60]. Within the realm of potential therapeutics for this condition, the inhibition of cholinesterase enzymes emerges as a pivotal target. Specifically, the AChE and BChE enzymes play essential roles in terminating cholinergic neurotransmission by facilitating the hydrolysis of acetylcholine. As disease progresses, acetylcholine levels decrease, and using AChE and BChE inhibitors has been shown to effectively relieve AD symptoms [61]. Notably, BChE enzyme levels exhibit an upward trend as the disease advances and in correlation with an individual’s age. Consequently, the exploration of selective AChE and BChE inhibitors holds great promise and has garnered significant attention from researchers. Additionally, it’s noteworthy that plant extract inhibitors are known to possess potent and undesirable side effects [62,63], highlighting one of the primary objectives of this investigation.

The evaluation of the inhibitory activity of *I. sarana* and its complex with β-CD on the enzymes acetylcholinesterase (AChE) and butyrylcholinesterase (BChE) has provided valuable insights into their potential as neuroprotective agents. The results reveal significant variations in inhibition values among the different extracts and the complex. Extracts obtained from ethanol, 70% ethanol, and ethyl acetate exhibited the highest values for AChE inhibition, with values of 3.42, 3.18, and 3.1 mg GALAE/g dry extract, respectively. For BChE inhibition, the ethanol-based extracts also displayed higher values, with 2.82 and 1.43 mg GALAE/g dry extract for the ethanol and ethyl acetate extracts, respectively. Conversely, the water extract showed low AChE inhibition activity (0.12 mg GALAE/g dry extract), while the complex with β-CD exhibited significant BChE inhibition activity (2.95 mg GALAE/g dry complex) (Table 3).

The observation of varying inhibition values among the different extracts underscores the significance of solvent choices in the final composition of the extracts. Extracts obtained from ethanol, 70% ethanol, and ethyl acetate demonstrated significant AChE inhibition activity, indicating the presence of compounds capable of modulating this enzyme closely linked to cholinergic neurotransmission [64,65,66]. These extracts exhibited higher inhibitory activity against AChE, potentially attributed to the presence of specific bioactive compounds that possess particular affinities for this enzyme.

The inhibition values for BChE also exhibited variations, with the ethanol extract showing noteworthy activity. Inhibition of BChE is associated with positive effects on cognitive function and the prevention of neurodegenerative diseases [67,68,69]. It is intriguing to note that the complex with β-CD displayed significant BChE inhibition activity, suggesting that complex formation might influence interactions between the extracted compounds and the target enzymes.

#### 3.5.3. Tyrosinase Inhibitory Activity

Tyrosinase, a copper-containing enzyme, plays a pivotal role in catalysing melanin synthesis through oxidation reactions, leading to undesired darkening resulting from the enzymatic oxidation of phenols [70]. This concern arises from its significant involvement not only in mammalian melanogenesis but also in enzymatic browning in fruits and fungi [71]. Consequently, the development of tyrosinase inhibitors holds broad applications, extending to medicinal [72] and cosmetic products [73], particularly in addressing hyperpigmentation issues. In light of this, downregulating or inhibiting tyrosinase activity has become a common approach, both in dealing with pigment disorders and as a whitening agent for aesthetic purposes serving as one of the focal points of this present study.

The evaluation of the inhibitory activity of *I. sarana* and its complex with β-CD on the enzyme tyrosinase has provided intriguing insights into their potential for melanogenesis regulation. The results unveiled significant differences in inhibition values among the various extracts and the complex. Extracts obtained from ethanol and 70% ethanol exhibited the highest tyrosinase inhibition values, with 27.87 mg KAE/g dry extract and 39.88 mg KAE/g dry extract, respectively (Table 3). The ethanol extract displayed remarkable inhibitory activity, suggesting the presence of compounds capable of modulating melanin production and potentially applicable in cosmetic or dermatological applications. The weakest tyrosinase inhibitory effect was found in the ethyl acetate extract with 10.32 mg KAE/g dry extract. Other extracts displayed varying inhibition values, emphasizing the impact of solvent choice on the enzyme tyrosinase activity.

The observation of variable inhibition values among the different extracts and the complex underscores the impact of solvent choice on the chemical composition of the extracts and, consequently, their capacity to modulate tyrosinase enzyme activity. The highest inhibitory activity was observed in the ethanol and 70% ethanol extracts, indicating that these solvents favored the extraction of potentially bioactive compounds capable of regulating melanin production [74,75,76]. This observation aligns with ethanol’s known properties to solubilize phenolic and flavonoid compounds, which are often associated with tyrosinase inhibitory activities.

The absence of inhibitory activity in the water extract raises questions about the active compounds responsible for this activity. This could be attributed to differences in solubility of bioactive compounds in water compared to organic solvents [77]. However, it is possible that the active compounds might be present in too low quantities in the water extract to generate observable inhibitory activity. The influence of the complex with β-CD on tyrosinase inhibitory activity was not extensively explored in this study, but it could be interesting for future research to investigate how complex formation might influence the regulation of melanin production.

Similar to antioxidant assays, we designed a circle heat map based on the enzyme inhibition results and the results are given in Figure 5B. In contrast to antioxidant ability, the extracts/complex based on the enzyme inhibitory were differently classified. This fact can be explained by different effects (synergetic or antagonistic) of chemicals on the enzymes and complex structures of active sites of each enzymes. As further insights, we examined the relationship between total bioactive compounds and enzyme inhibitory properties. In Figure 5C, we did not observe any correlation between total phenolic content and enzyme inhibitory properties (R < 0.5). However, AChE, amylase and glucosidase were moderately correlated with the total flavonoid content (R > 0.7).

When considering the collective findings, the observed inhibitory effect on enzymes in the tested samples can be attributed to the presence of specific compounds. For instance, chlorogenic acid has been recognized as a crucial inhibitor of both AChE and BChE, and its OH group, substituting quinic acid, can interact with the active sites of these enzymes, as detailed by Oboh et al. [78]. Likewise, the OH group participates in the formation of hydrogen bonds within the active site of amylase, as suggested by Zheng et al. [79]. Furthermore, compounds such as rutin [80], hyperoside [81], and hydroxybenzoic acid [82] are also known as significant inhibitors. Notably, the glycosylation and the number of hydroxyl groups within the flavonoid rings of their structure can impact their enzyme inhibitory effects, as discussed by Li et al. [83]. Additionally, partenolide has demonstrated the ability to enhance acetylcholine levels by inhibiting AChE activity in diabetic rats, as reported by Khare et al. [84].

## 4. Conclusions

The study findings showed that water and 70% ethanol extracts had the highest phenolic content, while ethyl acetate extracts contained notable flavonoids. This observation was further substantiated through the utilization of ESI MS/MS analysis. Moreover, the extracts of ethyl acetate, n-hexane, and DCM were effectively enclosed within an inclusion complex of β-CD, a fact that was verified by means of FTIR-ATR and SEM examinations.

It is noteworthy that the water-based extract and 70% ethanol extracts exhibited the highest level of antioxidant activity among all tested extract. This indicates a significant potential for these extracts to serve as potent sources of antioxidants, thereby holding promise for various applications in the realm of health and wellness.

Similarly, ethanol and n-hexane extracts displayed higher inhibition of the AChE and BChE, respectively. Moreover, the 70% ethanol extract exhibited elevated inhibition of the tyrosinase and α-glucosidase enzymes, and ethyl acetate demonstrated pronounced inhibition of the amylase enzyme. Notably, the inclusion complex displayed relatively less or no antioxidant efficacy and enzyme inhibitory potential compared to the unencapsulated extracts. This variation in inhibition values observed across the distinct extracts and the inclusion complex underscores the significant influence of solvent selection on the chemical composition of the extracts. Consequently, this choice of solvent directly affects their potential to effectively modulate enzyme activity. These findings emphasize the critical significance of comprehending the interplay between active compounds, solvents, and complex formations, as a means to enhance and optimize the antioxidant properties inherent in these extracts. The outcomes of this study offer invaluable insights for future investigations aimed at comprehending the fundamental mechanisms at play, thereby unlocking the potential of these extracts for applications that can distinctly contribute to enhancing human health and well-being.

## Figures and Tables

**Table 1:** Total phenolic and flavonoid content of the tested samples *.

Extracts	Total Phenolic Content (mg GAE/g Dry Extract or Complex)	Total Flavonoid Content (mg RE/g Dry Extract or Complex)
n-Hexane	12.45 ± 0.44 ^f^	4.60 ± 0.18 ^e^
Ethyl acetate	23.77 ± 0.15 ^d^	22.60 ± 0.44 ^a^
Dicholoromethane	20.19 ± 0.78 ^e^	16.57 ± 0.32 ^b,c^
Ethanol	38.42 ± 0.10 ^c^	17.23 ± 0.48 ^b^
70% Ethanol	46.36 ± 0.12 ^b^	15.93 ± 0.30 ^c^
Water	55.55 ± 0.42 ^a^	9.11 ± 0.14 ^d^
n-Hexane/β-CD	3.00 ± 0.03 ^h^	1.96 ± 0.26 ^f^
Ethyl acetate/β-CD	4.17 ± 0.11 ^g^	3.82 ± 0.41 ^e^
DCM/-β-CD	4.54 ± 0.09 ^g^	1.81 ± 0.07 ^f^

* Values are reported as mean ± SD of three parallel measurements. GAE: Gallic acid equivalents; RE: Rutin equivalents. Different letters (“^a^” indicates the highest level) in the same column indicate significant differences in the tested extracts and complexes (*p* < 0.05).

**Table 2:** Antioxidant properties of the tested samples *.

Extracts	DPPH (mg TE/g Dry Extract/Complex)	ABTS (mg TE/g Dry Extract/Complex)	CUPRAC (mg TE/g Dry Extract/Complex)	FRAP (mg TE/g Dry Extract/Complex)	PBD (mmol TE/g Dry Extract/Complex)	MCA (mg EDTAE/g Dry Extract/Complex)
n-Hexane	17.95 ± 0.24 ^e^	12.02 ± 0.62 ^e^	46.56 ± 0.71 ^e^	24.50 ± 1.87 ^d^	1.49 ± 0.03 ^b^	13.85 ± 0.56 ^c^
Ethyl acetate	27.95 ± 0.30 ^c^	53.24 ± 0.10 ^c^	71.51 ± 2.43 ^d^	33.25 ± 0.21 ^c^	2.07 ± 0.14 ^a^	15.62 ± 0.97 ^c^
Dicholoromethane	25.90 ± 0.24 ^d^	48.29 ± 0.37 ^d^	64.50 ± 2.94 ^d^	31.39 ± 0.36 ^c,d^	2.01 ± 0.23 ^a^	15.44 ± 1.09 ^c^
Ethanol	45.84 ± 0.19 ^b^	73.61 ± 0.74 ^b^	159.00 ± 1.30 ^c^	74.18 ± 0.39 ^b^	1.97 ± 0.02 ^a^	10.53 ± 0.41 ^d^
70% Ethanol	47.97 ± 0.02 ^a^	106.50 ± 0.09 ^a^	224.31 ± 0.02 ^a^	110.40 ± 3.96 ^a^	1.61 ± 0.06 ^b^	19.22 ± 0.82 ^b^
Water	47.63 ± 0.04 ^a^	106.41 ± 0.01 ^a^	193.85 ± 8.08 ^b^	113.88 ± 7.43 ^a^	1.28 ± 0.03 ^c^	22.99 ± 0.08 ^a^
n-Hexane/β-CD	15.17 ± 0.53 ^f^	na	9.98 ± 0.22 ^f^	7.47 ± 0.04 ^e^	0.12 ± 0.02 ^e^	0.96 ± 0.25 ^g^
Ethyl acetate/β-CD	13.98 ± 0.36 ^g^	1.52 ± 0.04 ^f^	10.67 ± 0.42 ^f^	8.11 ± 0.18 ^e^	0.15 ± 0.01 ^e^	1.87 ± 0.08 ^f^
DCM/β-CD	14.30 ± 0.55 ^f,g^	2.01 ± 0.36 ^f^	11.82 ± 0.20 ^f^	7.97 ± 0.05 ^e^	0.26 ± 0.02 ^d^	4.08 ± 0.53 ^e^

* Values are reported as mean ± SD of three parallel measurements. PBD: Phosphomolybdenum; MCA: Metal chelating Activity; TE: Trolox Equivalent; EDTAE: EDTA equivalent. Different letters (“^a^” indicates the strongest ability) in the same column indicate significant differences in the tested extracts and complexes (*p* < 0.05).

**Table 3:** Enzyme inhibitory effects of the tested extracts *.

Extracts	AChE (mg GALAE/g Dry Extract/Complex)	BChE (mg GALAE/g Dry Extract/Complex)	Tyrosinase (mg KAE/g Dry Extract/Complex)	Amylase (mmol ACAE/g Dry Extract/Complex)	Glucosidase (mmol ACAE/g Dry Extract/Complex)
n-Hexane	2.99 ± 0.06 ^c^	3.37 ± 0.31 ^a^	35.48 ± 2.19 ^b^	0.47 ± 0.01 ^a,b^	0.89 ± 0.01 ^b^
Ethyl acetate	3.17 ± 0.08 ^b,c^	1.43 ± 0.22 ^c^	10.32 ± 1.38 ^e^	0.49 ± 0.02 ^a^	0.80 ± 0.01 ^c^
DCM	2.69 ± 0.06 ^d^	2.37 ± 0.38 ^b^	19.57 ± 1.08 ^d^	0.45 ± 0.01 ^b,c^	0.90 ± 0.03 ^a,b^
Ethanol	3.42 ± 0.12 ^a^	2.82 ± 0.18 ^a,b^	27.87 ± 0.57 ^c^	0.44 ± 0.01 ^c^	0.88 ± 0.01 ^b^
70% Ethanol	3.18 ± 0.05 ^b^	0.07 ± 0.01 ^d^	39.88 ± 0.46 ^a^	0.33 ± 0.01 ^d^	0.93 ± 0.01 ^a^
Water	0.12 ± 0.06 ^e^	na	na	0.05 ± 0.01 ^f^	0.21 ± 0.01 ^d^
n-Hexane/β-CD	na	1.27 ± 0.23 ^c^	na	0.10 ± 0.01 ^e^	na
Ethyl acetate/β-CD	na	2.97 ± 0.06 ^a^	na	0.07 ± 0.01 ^e,f^	na
DCM/β-CD	0.16 ± 0.03 ^e^	2.95 ± 0.09 ^a,b^	na	0.09 ± 0.01 ^e^	na

* Values are reported as mean ± SD of three parallel measurements. GALAE: Galantamine equivalent; KAE: Kojic acid equivalent; ACAE: Acarbose equivalent; na: not active. Different letters (“^a^” indicates the strongest ability) in the same column indicate significant differences in the tested extracts and complexes (*p* < 0.05).

## Data Availability

Data is contained within the article and supplementary material.

## Supplementary Materials

The following supporting information can be downloaded at: https://www.mdpi.com/article/10.3390/antiox12101842/s1, Table S1: Specialized metabolites in *Inula sarana* extracts assayed by UHPLC-HRMS; Table S2: Specialized metabolites in *Inula sarana* extracts assayed by UHPLC-HRMS in positive ion mode; Table S3: The equations, range and R2 values of standard compounds in the biological activity assays; Figure S1: Extracted ion chromatograms of hydroxybenzoic and hydroxycinnamic acids and their derivatives; Figure S2: Extracted ion chromatograms of acylquinic acids; Figure S3: Extracted ion chromatograms of caffeoylhexaric acids; Figure S4. Extracted ion chromatograms of flavonoid glycosides; Figure S5: Extracted ion chromatograms of flavonoid aglycons. References [22,85] cited in the supplementary materials.
