# Supplementary material for: A Comparative Study on UHPLC-HRMS Profiles and Biological Activities of Inula sarana Different Extracts and Its Beta-Cyclodextrin Complex: Effective Insights for Novel Applications
Source: Antioxidants (Basel). 2023 Oct 10;12(10):1842. doi: 10.3390/antiox12101842 (PMC10604322; doi:10.3390/antiox12101842)
Supplement: Supplementary file 1 [file antioxidants-12-01842-s001.zip › antioxidants-2616709-supplementary.pdf]

# A Comparative Study on UHPLC-HRMS Profiles and Biological Activities of *Inula sarana* Different Extracts and Its Beta-Cyclodextrin Complex: Effective Insights for Novel Applications

**Gokhan Zengin [1,*], Nilofar [1,2], Evren Yildiztugay [3], Abdelhakim Bouyahya [4], Halit Cavusoglu [5], Reneta Gevrenova [6] and Dimitrina Zheleva-Dimitrova [6,*]**

1 Physiology and Biochemistry Laboratory, Department of Biology, Science Faculty, Selcuk University, Konya 42130, Turkey; nilofar.nilofar@unich.it

2 Department of Pharmacy, Botanic Garden "Giardino dei Semplici", Università degli Studi "Gabriele d'Annunzio", via dei Vestini 31, 66100 Chieti, Italy

3 Department of Biotechnology, Science Faculty, Selcuk University, Konya 42130, Turkey; eytugay@selcuk.edu.tr

4 Laboratory of Human Pathologies Biology, Faculty of Sciences, Mohammed V University in Rabat, Rabat 10106, Morocco; a.bouyahya@um5r.ac.ma

5 Department of Physics, Science Faculty, Selcuk University, Konya 42130, Turkey; hcavusoglu@selcuk.edu.tr

6 Department of Pharmacognosy, Faculty of Pharmacy, Medical University of Sofia, 1000 Sofia, Bulgaria; rgevrenova@pharmfac.mu-sofia.bg

* Correspondence: gokhanzengin@selcuk.edu.tr (G.Z.); dzheleva@pharmfac.mu-sofia.bg (D.Z.-D.)

## Supplementary Materials

*Assays for Total Phenolic and Flavonoid Contents*

The total phenolic content was determined by employing the methods given in the literature with some modification. Sample solution (0.25 mL) was mixed with diluted Folin–Ciocalteu reagent (1 mL, 1:9, v/v) and shaken vigorously. After 3 min, $Na_2CO_3$ solution (0.75 mL, 1%) was added and the sample absorbance was read at 760 nm after a 2 h incubation at room temperature. The total phenolic content was expressed as milligrams of gallic acid equivalents (mg GAE/g extract) (Uysal et al., 2017).

The total flavonoid content was determined using the $AlCl_3$ method. Briefly, sample solution (1 mL) was mixed with the same volume of aluminum trichloride (2%) in methanol. Similarly, a blank was prepared by adding sample solution (1 mL) to methanol (1 mL) without $AlCl_3$. The sample and blank absorbances were read at 415 nm after a 10 min incubation at room temperature. The absorbance of the

blank was subtracted from that of the sample. Rutin was used as a reference standard and the total flavonoid content was expressed as milligrams of rutin equivalents (mg RE/g extract) (Uysal et al., 2017).

*Determination of Antioxidant and Enzyme Inhibitory Effects*

Antioxidant (DPPH and ABTS radical scavenging, reducing power (CUPRAC and FRAP), phosphomolybdenum and metal chelating (ferrozine method)) and enzyme inhibitory activities (cholinesterase (Elmann's method), tyrosinase (dopachrome method), $\alpha$-amylase (iodine/potassium iodide method), $\alpha$ -glucosidase (chromogenic PNPG method) and pancreatic lipase (*p*-nitrophenyl butyrate (p-NPB) method) were determined using the methods previously described by Uysal et al. (Uysal et al., 2017) and Grochowski et al. (Grochowski et al., 2017)

For the DPPH (1,1-diphenyl-2-picrylhydrazyl) radical scavenging assay: Sample solution was added to 4 mL of a 0.004% methanol solution of DPPH. The sample absorbance was read at 517 nm after a 30 min incubation at room temperature in the dark. DPPH radical scavenging activity was expressed as milligrams of trolox equivalents (mg TE/g extract).

For ABTS (2,2′-azino-bis(3-ethylbenzothiazoline) 6-sulfonic acid) radical scavenging assay: Briefly, ABTS+ was produced directly by reacting 7 mM ABTS solution with 2.45 mM potassium persulfate and allowing the mixture to stand for 12–16 in the dark at room temperature. Prior to beginning the assay, ABTS solution was diluted with methanol to an absorbance of 0.700 ± 0.02 at 734 nm. Sample solution was added to ABTS solution (2 mL) and mixed. The sample absorbance was read at 734 nm after a 30 min incubation at room temperature. The ABTS radical scavenging activity was expressed as milligrams of trolox equivalents (mg TE/g extract).

For CUPRAC (cupric ion reducing activity) activity assay: Sample solution was added to premixed reaction mixture containing $CuCl_2$ (1 mL, 10 mM), neocuproine (1 mL, 7.5 mM) and $NH_4Ac$ buffer (1 mL, 1 M, pH 7.0). Similarly, a blank was prepared by adding sample solution (0.5 mL) to premixed reaction mixture (3 mL) without $CuCl_2$ . Then, the sample and blank absorbances were read at 450 nm after a 30 min incubation at room temperature. The absorbance of the blank was subtracted from that of the sample. CUPRAC activity was expressed as milligrams of trolox equivalents (mg TE/g extract).

For FRAP (ferric reducing antioxidant power) activity assay: Sample solution was added to premixed FRAP reagent (2 mL) containing acetate buffer (0.3 M, pH 3.6), 2,4,6-tris(2-pyridyl)-S-triazine (TPTZ) (10 mM) in 40 mM HCl and ferric chloride (20 mM) in a ratio of 10:1:1 (v/v/v). Then, the sample absorbance was read at 593 nm after a 30 min incubation at room temperature. FRAP activity was expressed as milligrams of trolox equivalents (mg TE/g extract).

For phosphomolybdenum method: Sample solution was combined with 3 mL of reagent solution (0.6 M sulfuric acid, 28 mM sodium phosphate and 4 mM ammonium molybdate). The sample absorbance was read at 695 nm after a 90 min incubation at 95 °C. The total antioxidant capacity was expressed as millimoles of trolox equivalents (mmol TE/g extract).

For metal chelating activity assay: Briefly, sample solution was added to $FeCl_2$ solution (0.05 mL, 2 mM). The reaction was initiated by the addition of 5 mM ferrozine (0.2 mL). Similarly, a blank was prepared by adding sample solution (2 mL) to $FeCl_2$ solution (0.05 mL, 2 mM) and water (0.2 mL) without ferrozine. Then, the sample and blank absorbances were read at 562 nm after 10 min incubation at room temperature. The absorbance of the blank was sub-tracted from that of the sample. The metal

chelating activity was expressed as milligrams of EDTA (disodium edetate) equivalents (mg EDTAE/g extract).

For Cholinesterase (ChE) inhibitory activity assay: Sample solution (was mixed with DTNB (5,5-dithio-bis(2-nitrobenzoic) acid, Sigma, St. Louis, MO, USA) (125 μL) and AChE (acetylcholines-terase (Electric ell acetylcholinesterase, Type-VI-S, EC 3.1.1.7,Sigma)), or BChE (butyrylcholinesterase (horse serum butyrylcholinesterase, EC 3.1.1.8, Sigma)) solution (25 μL) in Tris–HCl buffer (pH 8.0) in a 96-well microplate and incubated for 15 min at 25 °C. The reaction was then initiated with the addition of acetylthiocholine iodide (ATCI, Sigma) or butyrylthiocholine chloride (BTCl, Sigma) (25 μL). Similarly, a blank was prepared by adding sample solution to all reaction reagents without enzyme (AChE or BChE) solution. The sample and blank absorbances were read at 405 nm after 10 min incubation at 25 °C. The absorbance of the blank was subtracted from that of the sample and the cholinesterase inhibitory activity was expressed as galanthamine equivalents (mgGALAE/g extract).

For Tyrosinase inhibitory activity assay: Sample solution was mixed with tyrosinase solution (40 μL, Sigma) and phosphate buffer (100 μL, pH 6.8) in a 96-well microplate and incubated for 15 min at 25 °C. The reaction was then initiated with the addition of L-DOPA (40 μL, Sigma). Similarly, a blank was prepared by adding sample solution to all reaction reagents without enzyme (tyrosinase) solution. The sample and blank absorbances were read at 492 nm after a 10 min incubation at 25 °C. The absorbance of the blank was subtracted from that of the sample and the tyrosinase inhibitory activity was expressed as kojic acid equivalents (mgKAE/g extract).

For $\alpha$-amylase inhibitory activity assay: Sample solution was mixed with $\alpha$-amylase solution (ex-porcine pancreas, EC 3.2.1.1, Sigma) (50 μL) in phosphate buffer (pH 6.9 with 6 mM sodium chloride) in a 96-well microplate and incubated for 10 min at 37 °C. After pre-incubation, the reaction was initiated with the addition of starch solution (50 μL, 0.05%). Similarly, a blank was prepared by adding sample solution to all reaction reagents without enzyme ($\alpha$-amylase) solution. The reaction mixture was incubated 10 min at 37 °C. The reaction was then stopped with the addition of HCl (25 μL, 1 M). This was followed by addition of the iodine-potassium iodide solution (100 μL). The sample and blank absorbances were read at 630 nm. The absorbance of the blank was subtracted from that of the sample and the $\alpha$-amylase inhibitory activity was expressed as acarbose equivalents (mmol ACE/g extract).

For $\alpha$-glucosidase inhibitory activity assay: Sample solution was mixed with glutathione (50 μL), $\alpha$-glucosidase solution (from Saccharomyces cerevisiae, EC 3.2.1.20, Sigma) (50 μL) in phosphate buffer (pH 6.8) and PNPG (4-N-trophenyl-$\alpha$-D-glucopyranoside, Sigma) (50 μL) in a 96-well microplate and incubated for 15 min at 37 °C. Similarly, a blank was prepared by adding sample solution to all reaction reagents without enzyme ($\alpha$-glucosidase) solution. The reaction was then stopped with the addition of sodium carbonate (50 μL, 0.2 M). The sample and blank absorbances were read at 400 nm. The absorbance of the blank was subtracted from that of the sample and the $\alpha$-glucosidase inhibitory activity was expressed as acarbose equivalents (mmol ACE/g extract).

**Table S1.** Specialized metabolites in *Inula sarana* extracts.

| No. | Identified/tentatively annotated compound | Molecular formula | Exact mass [M-H]- | Fragmentation pattern in (-) ESI-MS/MS | $t_R$ (min) | Δppm | Distribution |
|---|---|---|---|---|---|---|---|
| | | | | **Hydroxybenzoic, hydroxycinnamic and acylquinic acids, and derivatives** | | | |
| **1** | Protocatechuic acid-*O*-hexoside | $C_{13}H_{16}O_9$ | 315.0722 | 315.0720 (2.9), 153.0181 (100), 109.0280 (51.4), 91.0176 (0.1), 81.0280 (51.4) | 1.19 | -0.135 | 2,3,4,5 |
| **2** | Protocatechuic acid-*O*-hexoside isomer | $C_{13}H_{16}O_9$ | 315.0722 | 315.0725 (100), 153.0182 (30.0), 109.0285 (9.8), 108.0202 (90.5) | 1.19 | -0.135 | 2,3,4,5 |
| **3** | Hydroxybenzoic acid-hexoside | $C_{13}H_{16}O_8$ | 299.0779 | 299.0776 (1.0), 137.0231 (100), 93.0330 (70.5) | 1.29 | 1.235 | 5,6 |
| **4** | Vanillic acid-*O*-hexoside | $C_{14}H_{18}O_9$ | 329.0890 | 329.0876 (1.9), 167.0339 (100), 152.0103 (24.0), 123.0437 (13.7), 108.0201 (38.5) | 1.78 | -0.776 | 2,4,5,6 |
| **5** | Protocatechuic acid[a] | $C_7H_6O_4$ | 153.0182 | 153.0182 (15.4), 109.0280 (100), 91.0173 (0.9), 81.0329 (1.3) | 2.04 | 0.211 | 2,4,5,6 |
| **6** | Syringic acid 4-*O*-hexoside | $C_{15}H_{20}O_{10}$ | 359.0984 | 359.0988 (7.7), 197.0447 (100), 182.0211 (19.3), 166.9976 (8.0), 153.0545 (15.1), 138.0309 (26.4), 123.0074 (33.0) | 2.28 | 1.198 | 2,4,5,6 |
| **7** | Neochlorogenic (3-caffeoylquinic) acid[a] | $C_{16}H_{18}O_9$ | 353.0867 | 353.0881 (38.6), 191.0553 (100), 179.0340 (60.0), 173.0448 (3.1), 161.0233 (3.7), 135.0438 (47.4), 127.0386 (1.9), 111.0439 (1.2), 93.0331 (4.3), 85.0280 (8.7) | 2.37 | 0.835 | 4,5,6 |
| **8** | Caffeic acid-*O*-hexoside | $C_{15}H_{18}O_9$ | 341.0867 | 341.0864 (4.1), 179.0340 (100), 161.0230 (2.5), 135.0438 (59.9), 117.0329 (0.4) | 2.43 | -4.032 | 4,5,6 |
| **9** | Caffeic acid-*O*-hexoside isomer | $C_{15}H_{18}O_9$ | 341.0867 | 341.0882 (1.5), 179.0339 (1.9), 135.0438 (100), 107.088 (0.8) | 2.57 | 1.245 | 5,6 |
| **10** | Gentisic acid-*O*-hexoside | $C_{13}H_{16}O_9$ | 315.0722 | 315.0726 (41.2), 153.0181 (86.6), 135.0072 (3.7), 109.0280 (100) | 2.58 | 0.415 | 4,5,6 |
| **11** | *p*-hydroxyphenylacetic acid-*O*-hexoside | $C_{14}H_{18}O_8$ | 313.0929 | 313.0928 (1.8), 151.0388 (100), 123.0079 (0.5), 109.0279 (3.6) | 2.68 | -0.386 | 5 |
| **12** | Vanillic acid[a] | $C_8H_8O_4$ | 167.0350 | 167.0339 (91.0), 152.0103 (8.1), 123.0437 (100), 108.0199 (5.7), 95.0486 (7.7) | 2.75 | -6.478 | 5 |
| **13** | Caffeoylgluconic acid | $C_{15}H_{18}O_{10}$ | 357.0827 | 357.0824 (11.4), 195.0502 (100), 179.0341 (44.5), 177.0397 (10.2), 135.0438 (42.1), 87.0072 (7.7), 59.0124 (1.1) | 2.82 | -0.812 | 5,6 |
| **14** | *O*-caffeoyl hexose | $C_{15}H_{18}O_9$ | 341.0867 | 341.0880 (19.9), 281.0668 (100), 251.0561 (54.1), 221.0451 (52.8), 179.0340 (93.6), 161.0233 (60.7), 135.0438 (71.2), 107.0487 (2.0) | 2.83 | 0.688 | 4,5 |
| **15** | 4-Hydroxybenzoic acid | $C_7H_6O_3$ | 137.0230 | 137.0231 (100), 108.0203 (8.6), 93.0331 (3.3) | 2.83 | -9.614 | 1,2,3,5,6,7,8,9 |
| **16** | 3-Hydroxybenzoic acid | $C_7H_6O_3$ | 137.0230 | 137.0230 (13.1), 108.0204 (0.6), 93.0330 (100) | 2.83 | -9.614 | 1,2,3,5,6,7,8,9 |
| **17** | Hydroxybenzoic acid-hexoside | $C_{13}H_{16}O_8$ | 299.0779 | 299.0784 (1.4), 137.0230 (100), 93.0330 (51.9) | 3.00 | 3.876 | 4,5,6 |

| | | | | | | | |
|---|---|---|---|---|---|---|---|
| **18** | Caffeoylgluconic acid isomer | $C_{15}H_{18}O_{10}$ | 357.0827 | 357.0818 (4.7), 195.0654 (100), 135.0438 (5.7), 59.0123 (12.2) | 3.41 | -2.604 | 5 |
| **19** | *O*-caffeoyl hexose isomer | $C_{15}H_{18}O_{9}$ | 341.0867 | 341.0878 (28.9), 281.0667 (71.9), 251.0560 (40.5), 221.0450 (37.8), 179.0340 (100), 161.0233 (42.9), 135.0438 (73.4), 107.0484 (0.6) | 3.12 | 0.600 | 4,5 |
| **20** | Quinic acid | $C_{7}H_{12}O_{6}$ | 191.0561 | 191.0553 (100), 173.0445 (1.8), 155.0339 (0.4), 127.0387 (3.7), 111.0437 (1.5), 93.0331 (6.8), 85.0280 (19.5) | 3.19 | -4.404 | 4,5,6 |
| **21** | Chlorogenic (5-caffeoylquinic) acid[a] | $C_{16}H_{18}O_{9}$ | 353.0867 | 353.0878 (4.0), 191.0553 (100), 179.0341 (1.4), 173.0448 (0.6), 161.0235 (1.6), 135.0438 (0.7), 127.0389 (2.0), 111.0437 (0.8), 93.0330 (3.1), 85.0279 (7.5) | 3.19 | -0.100 | 1,2,3,4,5,6 |
| **22** | *p*-coumaric acid[a] | $C_{9}H_{8}O_{3}$ | 163.0389 | 163.0388 (13.2), 135.0438 (8.5), 119.0488 (100) | 3.34 | -7.958 | 5 |
| **23** | 4-Caffeoylquinic acid | $C_{16}H_{18}O_{9}$ | 353.0867 | 353.0883 (31.7), 191.0554 (41.6), 179.0340 (66.2), 173.0445 (100), 161.0231 (2.7), 135.0439 (48.1), 127.0386 (1.5), 111.0437 (3.4), 93.0330 (20.6), 85.0279 (7.2) | 3.37 | 1.401 | 1,2,3,4,5,6 |
| **24** | Caffeic acid[a] | $C_{9}H_{8}O_{4}$ | 179.0338 | 179.0340 (19.0), 135.0438 (100), 117.0331 (0.8), 107.0487 (1.5) | 3.55 | -5.485 | 2,4,5,6 |
| **25** | Gentisic acid[a] | $C_{7}H_{6}O_{4}$ | 153.0182 | 153.0181 (77.3), 135.0074 (30.5), 109.0280 (100), 91.0174 (5.2), 81.0331 (0.5) | 3.86 | 0.141 | 4,5 |
| **26** | 5-*p*-Coumaroylquinic acid | $C_{16}H_{18}O_{8}$ | 337.0929 | 337.0934 (8.6), 191.0553 (100), 173.0445 (7.0), 163.0390 (5.8), 127.0387 (1.0), 119.0489 (4.0), 111.0437 (3.1), 93.0330 (15.1), 85.0279 (4.5) | 3.96 | 1.363 | 4,5,6 |
| **27** | *p*-Hydroxyphenylacetic acid | $C_{8}H_{8}O_{3}$ | 151.0401 | 151.0388 (100), 136.0153 (8.6), 123.0438 (78.8), 107.0486 (87.2) | 3.47 | -8.391 | 2,4,5 |
| **28** | 5-Feruloylquinic acid | $C_{17}H_{20}O_{9}$ | 367.1035 | 367.1039 (16.3), 193.0503 (5.4), 191.0553 (100), 173.0446 (11.6), 135.0392 (0.3), 134.0360 (10.1), 111.0437 (0.4), 93.0330 (25.6), 85.0279 (4.7) | 4.40 | 1.075 | 4,5,6 |
| **29** | 1-Caffeoyl-3-hydroxy-dihydrocaffeoylquinic acid | $C_{25}H_{26}O_{13}$ | 533.1301 | 533.1312 (28.3), 371.0987 (59.7), 353.0876 (5.2), 335.0779 (6.7), 335.0776 (2.9), 197.0447 (2.9), 191.0552 (14.7), 197.0448 (3.8), 179.0341 (13.7), 173.0444 (34.3), 161.0229 (5.0), 153.0544 (12.5), 135.0438 (100), 111.0435 (2.8), 93.0330 (10.0), 85.0279 (4.0) | 4.43 | 2.187 | 5,6 |
| **30** | *m*-Coumaric acid[a] | $C_{9}H_{8}O_{3}$ | 163.0389 | 163.0389 (8.9), 135.0438 (2.0), 119.0488 (100) | 4.56 | -1.127 | 5 |
| **31** | Syringic acid (caffeoyl)-hexoside | $C_{24}H_{26}O_{13}$ | 521.1301 | 521.1305 (83.4), 359.0974 (1.1), 323.0775 (20.2), 263.0554 (1.3), 203.0349 (0.7), 179.0340 (14.8), 161.0233 (54.2), 135.0438 (100) | 4.65 | 0.818 | 4,5,6 |
| **32** | Hydroxyisopropanoic acid-*O*-(coumaroyl)-hexoside | $C_{21}H_{32}O_{12}$ | 475.1821 | 475.1814 (47.8), 429.1766 (100), 325.1150 (3.0), 265.0935 (9.8), 235.0821 (1.6), 205.0708 (16.9), 163.0600 (56.5), 119.0336 (8.9) | 5.00 | -1.535 | 2,4,5,6 |

| | | | | | | | |
|---|---|---|---|---|---|---|---|
| **33** | Syringic acid (caffeoyl)-hexoside | $C_{24}H_{26}O_{13}$ | 521.1301 | 521.1303 (100), 359.0989 (5.4), 323.0774 (20.9), 203.0345 (0.6), 197.0448 (38.1), 182.0207 (4.4), 179.0341 (21.3), 166.9977 (3.4), 161.0232 (64.7), 151.0392 (0.7), 135.0438 (28.6), 123.0437 (9.0), 123.0077 (1.3) | 5.09 | 0.472 | 4,5,6 |
| **34** | Gentisic acid-(caffeoyl)-hexoside | $C_{22}H_{22}O_{12}$ | 477.1038 | 477.1042 (76.8), 323.0775 (44.3), 315.0722 (2.2), 221.0452 (5.2), 179.0340 (9.1), 161.0233 (45.2), 153.0182 (100),135.0439 (12.2), 133.0282 (16.2), 109.0280 (88.7) | 5.17 | 0.359 | 4,5,6 |
| **35** | Caffeic acid-(hydroxyisovaleryl)-hexoside | $C_{20}H_{26}O_{11}$ | 441.1402 | 441.1404 (78.7), 341.0864 (2.3), 323.0775 (11.1), 281.0667 (6.5), 251.0556 (4.4), 221.0457 (6.0), 179.0342 (15.4), 161.0232 (100), 135.0437 (18.4), 133.0281 (29.4), | 5.37 | 0.397 | 5,6 |
| **36** | Caffeic acid-*O*-(salicyl)-hexoside | $C_{22}H_{22}O_{11}$ | 461.1089 | 461.1099 (2.3), 323.0776 (47.3), 221.0454 (8.6), 179.0341 (18.5), 161.0233 (78.9), 137.0231 (40.7), 135.0438 (17.0), 133.0281 (32.2), 93.0330 (50.4) | 5.40 | 2.072 | 2,3,4,5,6 |
| **37** | Vanillic acid-4-*O*-(6-*O*-caffeoyl)-hexoside | $C_{23}H_{24}O_{12}$ | 491.1195 | 491.1197 (100), 323.0776 (35.2), 221.0452 (6.2), 179.0341 (17.4), 167.0339 (27.9), 161.0233 (66.1), 152.0103 (25.2), 135.0438 (21.8), 123.0438 (5.4), 108.0202 (15.2) | 5.52 | 0.388 | 4,5,6 |
| **38** | 3,4-Dicaffeoylquinic acid[a] | $C_{25}H_{24}O_{12}$ | 515.1195 | 515.1201 (77.6), 353.0882 (46.7), 335.0771 (10.4), 299.0554 (12.7), 227.0715 (2.0), 203.0344 (45.4), 191.0555 (33.8), 179.0341 (65.6), 173.0446 (100), 161.0234 (11.9), 135.0439 (75.0), 111.0436 (6.5), 93.0330 (28.7), 85.0279 (3.2) | 5.59 | 0.137 | 4,5,6 |
| **39** | 1,5-Dicaffeoylquinic acid[a] | $C_{25}H_{24}O_{12}$ | 515.1195 | 515.1198 (100), 353.0883 (16.5), 335.0791 (6.6), 191.0552 (31.2), 179.0340 (56.6), 173.0445 (58.9), 161.0233 (16.5), 135.0438 (54.4), 111.0435 (3.3), 93.0330 (15.6), 85.0280 (4.6) | 5.70 | 0.584 | 1,2,3,4,5,6 |
| **40** | 3,5-Dicaffeoylquinic acid[a] | $C_{25}H_{24}O_{12}$ | 515.1195 | 515.1205 (17.0), 353.0881 (87.0), 191.0553 (100), 179.0304 (48.1), 161.0231 (5.1), 111.0434 (2.0), 93.0333 (4.0), 85.0280 (6.6) | 5.85 | 1.904 | 1,2,3,4,5,6 |
| **41** | 4,5-dicaffeoylquinic acid | $C_{25}H_{24}O_{12}$ | 515.1195 | 515.1201 (75.6), 353.0882 (56.3), 203.0342 (3.8), 191.0553 (37.3), 179.0340 (65.6), 173.0445 (100), 135.0438 (76.4), 127.0385 (2.8), 111.0436 (4.9), 593.0330 (25.2), 85.0280 (5.6) | 6.22 | 1.089 | 1,2,3,4,5,6 |
| **42** | Salicylic acid[a] | $C_7H_6O_3$ | 137.0230 | 1537.0231 (9.8), 108.0203 (1.5), 93.0330 (100) | 6.28 | -9.611 | 1,3,4,5,6,7,8,9 |
| **43** | Caffeoyl-(salicyl)-hexoside isomer | $C_{22}H_{22}O_{11}$ | 461.1089 | 461.1091 (45.5), 323.0776 (28.4), 221.0448 (2.1), 179.0342 (5.4), 161.0234 (29.2), 137.0231 (100), 133.0281 (12.8), 135.0437 (7.3), 93.0331 (58.7) | 6.58 | 0.359 | 5 |

| | | | | | | | |
|---|---|---|---|---|---|---|---|
| **44** | 3-Caffeoyl-5-*p*-coumaroylquinic acid | $C_{25}H_{24}O_{11}$ | 499.1246 | 499.1256 (17.2), 353.0880 (54.1), 337.0930 (23.9), 191.0552 (100), 179.0338 (8.7), 173.0446 (17.5), 163.0389 (25.8), 161.0231 (8.1), 135.0439 (7.6), 119.0487 (19.5), 111.0437 (6.8), 93.0330 (12.2), 85.0280 (6.7) | 6.50 | 0.351 | 4,5 |
| **45** | 4-*p*-Coumaroyl-5-caffeoylquinic acid | $C_{25}H_{24}O_{11}$ | 499.1246 | 499.1244 (24.5), 353.0884 (6.5), 337.0933 (61.2), 191.0554 (13.2), 179.0341 (2.2), 173.0445 (100), 163.0390 (19.4), 127.0389 (0.7), 111.0438 (4.4), 93.0330 (23.5), 85.0279 (2.1) | 6.92 | -0.330 | 4,5 |
| **46** | 3,4,5-Tricaffeoylquinic acid | $C_{34}H_{30}O_{15}$ | 677.1512 | 677.1517 (100). 515.1199 (42.6), 353.0882 (47.7), 335.0778 (0.8), 203.0350 (0.9), 191.0554 (47.9), 179.0341 (68.0), 173.0446 (92.1), 161.0233 (25.3), 135.0439 (81.2), 111.0437 (5.6), 93.0331 (25.1), 85.0278 (4.3) | 7.77 | 0.793 | 5 |
| | | | | **Caffeoylhexaric acids** | | | |
| **47** | Caffeoylhexaric acid | $C_{15}H_{16}O_{11}$ | 371.0620 | 371.0611 (3.6), 209.0296 (100), 191.0189 (20.5), 179.0338 (1.5), 173.0088 (0.5), 147.0285 (3.7), 135.0436 (3.4), 129.0180 (3.7), 111.0073 (1.4), 85.0279 (42.4) | 1.31 | 0.150 | 5,6 |
| **48** | Caffeoylhexaric acid isomer | $C_{15}H_{16}O_{11}$ | 371.0620 | 371.0610 (3.6), 209.0296 (100), 191.0189 (24.0), 179.0338 (0.7), 173.0075 (1.5), 147.0285 (3.0), 135.0435 (2.2), 129.0180 (3.9), 111.0072 (1.7), 85.0279 (45.7) | 2.02 | -2.653 | 4,5,6 |
| **49** | Caffeoylhexaric acid isomer | $C_{15}H_{16}O_{11}$ | 371.0620 | 371.0639 (2.9), 209.0296 (100), 191.0188 (22.2), 179.0339 (0.6), 173.0083 (0.8), 147.0290 (3.5), 135.0437 (0.9), 129.0179 (4.3), 111.0071 (1.7), 85.0279 (37.6) | 2.43 | 5.243 | 4,5,6 |
| **50** | Caffeoylhexaric acid isomer | $C_{15}H_{16}O_{11}$ | 371.0620 | 371.0620 (3.8), 209.0297 (100), 191.0190 (21.7), 179.0343 (2.2), 147.0290 (1.8), 135.0442 (4.5), 129.0178 (2.5), 111.0074 (2.2), 85.0279 (38.0) | 2.67 | -0.012 | 5,6 |
| **51** | Dicaffeoylhexaric acid | $C_{24}H_{22}O_{14}$ | 533.0937 | 533.0928 (11.3), 371.0624 (95.2), 353.0526 (1.5), 209.0297 (100), 191.0190 (29.1), 179.0347 (3.6), 173.0080 (1.0), 147.0287 (4.5), 135.0438 (5.7), 5129.0178 (5.3), 111.0072 (3.5), 85.0279 (54.8), | 3.77 | -1.704 | 5,6 |
| **52** | Dicaffeoylhexaric acid isomer | $C_{24}H_{22}O_{14}$ | 533.0937 | 533.0879 (11.6), 371.0622 (79.9), 353.0491 (1.6), 335.0787 (0.4), 209.0297 (100), 191.0188 (23.6), 179.0341 (6.7), 161.0230 (0.8), 147.0284 (4.4), 135.0438 (9.8), 129.0178 (8.0), 85.0278 (53.1) | 4.12 | -10.858 | 5,6 |
| **53** | Dicaffeoylhexaric acid isomer | $C_{24}H_{22}O_{14}$ | 533.0937 | 533.0975 (11.4), 371.0612 (67.6), 353.0490 (3.5), 209.0297 (100), 191.0189 (27.6), 173.0343 (35.0), 147.0286 (4.3), 135.0438 (29.0), 129.0179 (7.0), 111.0076 (4.0), 85.0279 (50.5) | 4.70 | -3.905 | 5,6 |

| | | | | | | | |
|---|---|---|---|---|---|---|---|
| **54** | Dicaffeoylhexaric acid isomer | $C_{24}H_{22}O_{14}$ | 533.0937 | 533.0974 (9.3), 371.0618 (71.1), 353.0514 (3.0), 209.0297 (100), 191.0190 (22.7), 173.0445 (5.3), 161.0234 (2.2), 147.0287 (3.7), 135.0438 (7.2), 129.0184 (4.5), 111.0072 (3.0), 85.0279 (47.2) | 4.88 | -13.485 | 5,6 |
| **55** | Dicaffeoylhexaric acid isomer | $C_{24}H_{22}O_{14}$ | 533.0937 | 533.0895 (10.6), 371.0622 (82.6), 353.0504 (2.5), 179.0342 (2.9), 161.0231 (1.0), 209.0279 (100), 191.0190 (24.90), 147.0284 (4.0), 135.0439 (7.3), 129.0180 (4.5), 111.0073 (3.7), 85.0279 (48.3) | 5.16 | | 5,6 |
| **56** | Tetracaffeoylhexaric acid | $C_{42}H_{34}O_{20}$ | 857.1571 | 857.1796 (48.2), 695.1470 (57.2), 533.1164 (13.2), 371.0606 (2.9), 209.0297 (100), 191.0189 (46.5), 179.0340 (0.5), 173.0080 (2.3), 161.0230 (7.8), 147.0287 (9.4), 135.0438 (34.4), 129.0179 (20.3), 111.0074 (5.7), 85.0279 (71.4) | 5.21 | 1.673 | 5 |
| **57** | Tricaffeoylhexaric acid | $C_{33}H_{28}O_{17}$ | 695.1254 | 695.1260 (27.7), 533.0943 (32.1), 371.0623 (17.2), 353.0498 (1.5), 209.0297 (100), 191.0189 (15.8), 179.0340 (3.9), 173.0079 (1.1), 161.0230 (0.5), 147.0285 (3.2), 135.0438 (7.1), 129.0179 (5.8), 111.0076 (2.6), 85.0279 (39.4) | 5.89 | 0.874 | 4,5,6 |
| **58** | Tricaffeoylhexaric acid isomer | $C_{33}H_{28}O_{17}$ | 695.1254 | 695.1265 (26.4), 533.0944 (37.2), 371.0623 (18.8), 209.0296 (100), 191.0189 (19.3), 179.0337 (3.3), 173.0087 (1.1), 161.0235 (3.0), 147.0284 (5.4), 135.0440 (5.7), 129.0181 (5.7), 111.0072 (3.5), 85.0279 (43.4) | 6.33 | 1.665 | 5,6 |
| **59** | Tricaffeoylhexaric acid isomer | $C_{33}H_{28}O_{17}$ | 695.1254 | 695.1255 (28.0), 533.0934 (31.7), 371.0620 (17.0), 209.0296 (100), 191.0188 (16.0), 179.0341 (4.2), 173.0447 (1.5), 161.0233 (2.0), 147.0283 (3.7), 135.0437 (7.2), 129.0179 (6.5), 111.0073 (1.9), 85.0279 (41.8) | 6.46 | 0.255 | 5,6 |
| **60** | Tetracaffeoylhexaric acid isomer | $C_{42}H_{34}O_{20}$ | 857.1571 | 857.1584 (40.0), 695.1299 (8.6), 533.0965 (4.2), 371.0623 (13.8), 209.0297 (100), 191.0188 (15.7), 179.0340 (3.8), 173.0086 (0.9), 161.0233 (7.1), 147.0283 (4.1), 129.0181 (6.1), 111.0073 (1.4), 85.0279 (43.3) | 7.48 | 1.544 | 4,5,6 |
| **61** | Isobutanyl-tricaffeoylhexaric acid-hexoside | $C_{43}H_{44}O_{23}$ | 927.2201 | 927.2217 (100), 765.1890 (71.1), 603.1569 (18.8), 441.1046 (0.6), 423.0937 (1.8), 341.0883 (24.4), 323.0780 (10.0), 279.0721 (42.8), 261.0617 (13.9), 179.0340 (58.7), 173.0081 (5.6), 161.0233 (20.7), 147.0285 (6.4), 135.0438 (79.8), 129.0180 (18.3), 111.0072 (8.8) | 7.72 | 1.811 | 4,5,6 |
| **62** | 2-Methylbutanyl/isovaleryl-dicaffeoylhexaric acid | $C_{29}H_{30}O_{15}$ | 617.1512 | 617.1533 (14.9), 455.1205 (100), 293.0881 (46.8), 191.0188 (56.6), 179.0336 (4.1), 147.0288 (15.8), 135.0438 (14.2), 129.0178 (18.2), 111.0072 (5.1), 85.0279 (95.5) | 7.91 | 3.446 | 5,6 |

| | | | | | | | |
|---|---|---|---|---|---|---|---|
| **63** | 2-Methylbutanyl/isovaleryl-dicaffeoylhexaric acid isomer | $C_{29}H_{30}O_{15}$ | 617.1512 | 617.1553 (14.9), 455.1199 (100), 293.0881 (44.6), 191.0190 (58.0), 179.0336 (12.3), 147.0288 (13.0), 135.0438 (22.2), 129.0181 (12.6), 111.0073 (3.4), 85.0279 (80.8) | 8.22 | -2.862 | 5 |
| **64** | 2-Methylbutanyl/isovaleryl-tricaffeoylhexaric acid-hexoside | $C_{44}H_{46}O_{23}$ | 941.2357 | 941.2369 (100), 779.2042 (63.1), 617.1731 (18.5), 455.1232 (2.1), 341.0880 (25.0), 323.0880 (8.5), 293.0880 (35.5), 191.0190 (41.6), 179.0340 (63.8), 161.0233 (17.1), 147.0285 (6.6), 135.0438 (73.5), 129.0180 (17.9), 85.0279 (84.4) | 8.32 | 1.264 | 5 |
| **65** | Isobutanyl-tricaffeoylhexaric acid | $C_{37}H_{34}O_{18}$ | 765.1672 | 765.1685 (45.2), 603.1365 (46.8), 441.1043 (31.6), 279.0722 (86.2), 191.0189 (64.7), 179.0338 (10.4), 173.0077 (5.3), 161.0233 (6.7), 147.0286 (10.8), 135.0439 (26.9), 129.0180 (12.4), 111.0073 (5.8), 85.0279 (100) | 8.52 | 1.598 | 4,5,6 |
| **66** | 2-Methylbutanyl/isovaleryl-tricaffeoylhexaric acid | $C_{38}H_{36}O_{18}$ | 779.1829 | 779.1848 (45.6), 617.1522 (34.2), 455.1199 (26.3), 293.0882 (68.5), 191.0189 (61.9), 179.0340 (12.1), 173.0079 (4.1), 161.0233 (7.7), 147.0287 (11.2), 135.0438 (24.1), 129.0180 (14.4), 111.0075 (6.5), 85.0279 (100) | 9.15 | 2.391 | 4,5,6 |
| **67** | 2-Methylbutanyl/isovaleryl-tricaffeoylhexaric acid isomer | $C_{38}H_{36}O_{18}$ | 779.1829 | 779.1839 (46.2), 617.1521 (38.7), 455.1202 (29.3), 293.0880 (74.5), 191.0189 (67.8), 179.0339 (14.2), 173.0083 (5.1), 161.0235 (7.3), 147.0287 (13.3), 135.0438 (24.2), 129.0179 (14.0), 85.0279 (100) | 9.22 | 1.300 | 4,5,6 |
| | | | | **Flavonoids** | | | |
| **68** | Quercetin 7-*O*-rutinoside | $C_{27}H_{30}O_{16}$ | 609.1461 | 609.1467 (81.4), 301.0356 (100), 283.0255 (1.4), 271.0255 (0.5), 255.0293 (2.8), 237.0195 (0.8), 227.0341 (2.3), 133.0287 (2.7) | 4.64 | 0.824 | 2,3,4,5,6 |
| **69** | Rutin[a] | $C_{27}H_{30}O_{16}$ | 609.1461 | 609.1467 (100), 301.0353 (36.4), 300.0278 (68.6), 271.0249 (31.0), 255.0299 (14.5), 243.0299 (8.1), 178.9975 (3.0), 163.0030 (1.8), 151.0027 (5.4), 121.0285 (1.7), 107.0127 (2.1) | 5.09 | -0.879 | 1,2,3,4,5,6 |
| **70** | Eryodictiol *O*-rutinoside | $C_{27}H_{32}O_{15}$ | 595.1668 | 595.1677 (64.8), 287.0563 (75.5), 269.0434 (0.7), 175.0022 (4.0), 151.0025 (100), 135.0438 (54.3), 125.0227 (4.4), 107.0124 (19.1) | 5.11 | 1.372 | 4,5,6 |
| **71** | Isoquercitrin[a] | $C_{21}H_{20}O_{12}$ | 463.0882 | 463.0887 (100), 301.0346 (35.6), 300.0278 (79.7), 271.0250 (36.7), 255.0296 (14.1), 243.0299 (9.2), 227.0349 (2.9), 178.9982 (2.6), 161.0232 (2.4), 151.0027 (6.4), 121.0285 (1.5), 107.0126 (2.3) | 5.18 | 1.081 | 1,2,3,4,5,6 |
| **72** | Luteolin 7-*O*-neohesperidoside/rutinoside | $C_{27}H_{30}O_{15}$ | 593.1512 | 593.1521 (75.1), 285.0406 (100), 256.0368 (0.6), 239.0346 (0.7), 217.0513 (0.7), 199.0394 (1.8), 151.0023 (2.9), 133.0281 (4.2), 107.0124 (1.4) | 5.21 | 1.529 | 2,4,5,6 |

| | | | | | | | |
|---|---|---|---|---|---|---|---|
| **73** | Patuletin *O*-rutinoside | $C_{28}H_{32}O_{17}$ | 639.1567 | 639.1574 (100), 331.0457 (32.0), 330.0385 (67.5), 316.00224 (11.0), 315.0151 (20.4), 287.0201 (21.7), 271.0250 (4.7), 259.0247 (4.6), 243.0300 (3.8), 231.0297 (4.3), 215.0345 (4.5), 175.0028 (4.4), 165.9899 (4.6), 139.0025 (2.6), 136.9864 (1.0) | 5.23 | 1.154 | 1,2,3,4,5,6 |
| **74** | Hyperoside[a] | $C_{21}H_{20}O_{12}$ | 463.0882 | 463.0888 (100), 301.0353 (45.3), 300.0278 (81.8), 271.0250 (34.8), 255.0297 (15.9), 243.0297 (10.3), 227.0343 (2.1), 199.0389 (0.9), 178.9982 (3.4), 163.0023 (1.1), 151.0024 (5.9), 121.0278 (0.8), 107.0123 (2.2) | 5.28 | 1.254 | 1,2,3,4,5,6 |
| **75** | Luteolin 7-*O*-glucoside[a] | $C_{21}H_{20}O_{11}$ | 447.0933 | 447.0934 (100), 285.0404 (91.2), 256.0375 (4.7), 227.0347 (2.1), 211.0394 (1.0), 199.0396 (1.7), 175.0388 (1.4), 151.0024 (5.2), 133.0281 (4.8), 107.0124 (3.5) | 5.39 | 0.348 | 2,4,5 |
| **76** | Patuletin *O*-hexoside | $C_{22}H_{21}O_{13}$ | 493.0988 | 493.0993 (100), 331.0457 (30.5), 330.0385 (62.0), 315.0152 (20.2), 287.0200 (20.1), 271.0247 (4.4), 259.0242 (3.7), 243.0303 (2.8), 199.0912 (1.3), 187.0393 (2.2), 175.0026 (2.6), 165.09899 (3.6), 139.0024 (2.7) | 5.44 | 1.087 | 2,3,4,5,6 |
| **77** | Quercetin 3-*O*-acetylhexoside | $C_{23}H_{22}O_{13}$ | 505.0995 | 505.0995 (100), 463.0868 (1.7), 301.0350 (38.0), 300.0277 (96.2), 271.0250 (45.7), 255.0299 (18.1), 243.0298 (10.6), 227.03406 (2.9), 178.9974 (2.7), 163.0032 (1.9), 151.0025 (6.5), 121.0283 (1.3), 107.01234 (2.2) | 5.60 | 1.378 | 4,5,6 |
| **78** | Kaempferol 3-*O*-rutinoside[a] | $C_{27}H_{30}O_{15}$ | 593.1512 | 593.1520 (100), 285.0404 (85.0), 284.0329 (47.6), 255.0404 (38.5), 227.0347 (27.5), 211.0401 (2.5), 151.0023 (0.9), 135.0071 (0.9), 107.0129 (1.1) | 5.63 | 1.360 | 2,3,4,5,6 |
| **79** | Nepetin *O*-glucoside[a] | $C_{22}H_{22}O_{12}$ | 477.1038 | 477.1041 (100), 315.0489 (15.6), 300.0282 (9.6), 299.0201 (14.6), 285.0395 (1.7), 271.0249 (2.3), 255.0311 (0.5), 243.0296 (2.0), 227.0338 (0.9), 133.0283 (15.4) | 5.66 | 0.526 | 2,4,5,6 |
| **80** | Nepetin *O*-rutinoside | $C_{28}H_{32}O_{16}$ | 623.1618 | 623.1627 (100), 315.0513 (49.7), 300.0278 (27.9), 271.0252 (31.7), 243.0298 (16.6), 255.0302 (3.9), 227.0343 (2.0), 215.0349 (6.7), 165.9896 (5.0), 133.0283 (1.9) | 5.77 | 1.544 | 2,3,4,5,8 |
| **81** | Kaempferol 3-*O*-glucoside[a] | $C_{21}H_{20}O_{11}$ | 447.0932 | 447.0936 (100), 285.0398 (25.1), 284.0328 (57.7), 255.0299 (42.3), 227.0346 (42.8), 211.0402 (1.8), 151.0028 (2.7), 107.0125 (0.6) | 5.86 | 0.706 | 2,4,5 |
| **82** | Isorhamnetin 3-*O*-glucoside[a] | $C_{22}H_{22}O_{12}$ | 477.1042 | 477.1042 (100), 315.0508 (25.6), 314.0437 (41.9), 300.0276 (16.2), 299.0199 (18.0), 271.0250 (28.7), 255.0303 (2.7), 243.0297 (13.8), 215.0348 (7.2), 199.0392 (2.3), 178.9978 (1.4), 151.0033 (0.4), 133.0282 (2.4) | 6.03 | 0.253 | 2,5 |

| | | | | | | | |
|---|---|---|---|---|---|---|---|
| **83** | Chrysoeriol *O*-hexoside | $C_{22}H_{22}O_{11}$ | 461.1089 | 461.1096 (100), 446.0861 (22.6), 299.0560 (9.2), 284.0316 (5.8), 283.0251 (18.2), 255.0299 (54.5), 227.0346 (0.6), 163.0026 (0.8), 151.0024 (0.7) | 6.29 | 1.356 | 2,4,5 |
| **84** | Cirsiliol *O*-hexoside | $C_{23}H_{24}O_{12}$ | 491.1195 | 491.1200 (100), 329.0670 (9.1), 313.0358 (76.7), 299.0195 (2.3), 270.0172 (39.6), 298.0123 (11.8), 257.0086 (3.0), 242.0219 (2.5), 229.0141 (0.8), 136.9871 (1.3), 133.0283 (0.3) | 6.92 | 1.020 | 2,3,4,5,8 |
| **85** | Spinacetin | $C_{17}H_{14}O_8$ | 345.0616 | 345.0617 (99.4), 330.0384 (100), 315.0150 (45.8), 287.0201 (10.9), 271.0246 (1.5), 259.0248 (3.1), 243.0298 (2.7), 231.0297 (5.2), 215.0344 (3.1), 165.9897 (6.3), 149.0233 (9.6), 139.0388 (4.0), 136.9865 (2.1) | 7.23 | 0.346 | 2,4,5 |
| **86** | Luteolin[a] | $C_{15}H_{10}O_6$ | 285.0405 | 285.0406 (100), 267.0278 (0.2), 241.0511 (0.6), 217.0503 (1.0), 199.0389 (1.8), 151.0027 (4.6), 133.0282 (22.4), 121.0280 (0.7), 107.0123 (3.6) | 7.57 | 0.346 | 2,4,5 |
| **87** | Quercetin[a] | $C_{15}H_{10}O_7$ | 301.0354 | 301.0354 (100), 273.0411 (3.08), 257.0453 (1.24), 229.0506 (0.91), 178.9979 (21.66), 151.0024 (48.25), 121.0280 (13.33), 107.0123 (15.03) | 7.59 | 0.180 | 2,4,5 |
| **88** | Patuletin (6-methoxyquercetin) | $C_{16}H_{12}O_8$ | 331.0464 | 331.0463 (100), 316.0227 (64.3), 287.0198 (11.2), 271.0249 (6.0), 259.0251 (3.0), 243.0305 (2.6), 181.0133 (5.9), 165.9897 (18.0), 139.0025 (11.3), 136.9868 (2.0), 121.0282 (2.9) | 7.70 | 1.086 | 2,3,4,5 |
| **89** | Nepetin (6-methoxyluteolin) | $C_{16}H_{12}O_7$ | 315.0514 | 315.0513 (84.4), 300.0277 (100), 271.0250 (0.6), 255.0302 (1.3), 243.0296 (1.8), 227.0344 (1.6), 201.0189 (3.4), 165.9899 (1.4), 136.9868 (9.0), 109.9994 (0.6) | 7.74 | 0.965 | 2,3,4,5 |
| **90** | Axillarin | $C_{17}H_{14}O_8$ | 345.0616 | 345.0618 (98.7), 330.0384 (100), 315.0150 (48.8), 287.0199 (13.2), 271.0233 (0.4), 259.0250 (3.6), 243.0298 (3.2), 231.0293 (5.1), 215.0345 (4.8), 175.0023 (3.7), 165.9894 (6.3), 149.0233 (11.3), 139.0389 (5.1), 136.9864 (0.7), 109.9993 (3.8) | 8.25 | 0.172 | 2,3,4,5,8 |
| **91** | Cirsiliol | $C_{17}H_{14}O_7$ | 329.0677 | 329.0670 (77.2), 314.0437 (100), 299.0198 (54.3), 271.0248 (12.4), 255.0311 (0.6), 243.0302 (3.1), 227.0355 (2.6), 215.0345 (11.6), 199.0397 (1.3), 165.9898 (10.1), 136.9865 (2.8), 133.0282 (5.2), 109.9995 (6.1) | 8.38 | 0.985 | 1,2,3,4,5,7,8,9 |
| **92** | Naringenin | $C_{15}H_{12}O_5$ | 271.0612 | 271.0614 (100), 227.0716 (1.0), 177.0183 (10.4), 151.0025 (69.6), 125.0232 (0.7), 119.0488 (55.8), 107.0124 (17.2) | 8.58 | -0.468 | 2,3,4,5 |
| **93** | Apigenin[a] | $C_{15}H_{10}O_5$ | 269.0457 | 269.0456 (100), 225.0556 (1.7), 201.0556 (0.7), 151.0025 (5.3), 117.0331 (18.4), 107.0124 (4.8) | 8.62 | 0.050 | 2,4,5 |

| | | | | | | | |
|---|---|---|---|---|---|---|---|
| **94** | Quercetagetin-3,6,3'(4')-trimethyl ether | $C_{18}H_{16}O_8$ | 359.0772 | 359.0776 (100), 344.0540 (97.5), 329.0305 (82.6), 314.0065 (2.7), 301.0355 (3.1), 286.0121 (11.2), 258.018 (10.0), 230.0221 (6.3), 214.0262 (2.5), 165.9897 (7.0), 164.9818 (10.7), 163.0395 (2.9), 149.0230 (2.8), 148.0152 (2.2), 136.9867 (2.7), 109.9994 (5.9) | 8.69 | 1.029 | 1,2,3,4,5,8,9 |
| **95** | Kaempferol[a] | $C_{15}H_{10}O_6$ | 285.0405 | 285.0404 (100), 257.0450 (0.7), 239.0347 (0.9), 227.0350 (0.8), 211.0400 (1.1), 178.9920 (0.1), 151.0025 (1.7), 107.0123 (1.1) | 8.80 | 0.031 | 2,4,5 |
| **96** | Hispidulin (scutellarein-6-methyl ether)[a] | $C_{16}H_{12}O_6$ | 299.0563 | 299.0562 (58.8), 284.0327 (100), 255.0309 (0.9), 227.0348 (2.6), 211.0396 (2.1), 165.9897 (0.3), 139.0023 (0.7), 136.9867 (12.3), 109.9996 (0.3) | 8.84 | 0.230 | 1,2,3,4,5,8,9 |
| **97** | Chrysoeriol[a] | $C_{16}H_{12}O_6$ | 299.0562 | 299.0562 (100), 284.0327 (87.4), 256.0376 (19.5), 227.0349 (2.9), 211.0388 (0.4), 151.0026 (3.0), 107.0126 (2.1) | 8.93 | 0.330 | 1,2,3,4,5,8,9 |
| **98** | 6-Methoxykaempferol | $C_{16}H_{12}O_7$ | 315.0512 | 315.0514 (100), 300.0278 (63.2), 271.0250 (42.3), 255.0297 (9.4), 243.0298 (39.0), 227.0344 (2.0), 181.0134 (2.1), 165.9898 (9.6), 139.0022 (3.3), 136.9868 (1.7), 109.9995 (5.4) | 8.96 | 1.156 | 1,2,3,4,5,8,9 |
| **99** | Isorhamnetin[a] | $C_{16}H_{12}O_7$ | 315.0512 | 315.0514 (100), 300.0277 (50.0), 271.0249 (8.1), 227.0341 (1.1), 151.0023 (6.3), 107.0123 (8.1), 63.0224 (5.5) | 9.11 | 1.060 | 1,2,3,4,5,8 |
| **100** | Jaceosidin (6-hydroxyluteolin-6,3'-dimethyl ether)[a] | $C_{17}H_{14}O_7$ | 329.0677 | 329.0671 (74.8), 314.0436 (100), 299.0200 (48.4), 271.0250 (18.6), 255.0305 (0.9), 243.0297 (4.4), 227.0351 (2.6), 215.0343 (12.2), 165.9898 (10.0), 136.9857 (1.9), 133.0280 (6.5), 109.9995 (5.3) | 9.50 | 1.289 | 1,2,3,4,5,6,7,8,9 |
| **101** | Quercetagetin-3,6,3'(4')-trimethyl ether | $C_{18}H_{16}O_8$ | 359.0772 | 359.0776 (100), 344.0540 (97.5), 329.0305 (82.6), 314.0065 (2.7), 301.0355 (3.1), 286.0121 (11.2), 258.0168 (10.0), 230.0221 (6.3), 214.0262 (2.5), 165.9897 (7.0), 164.9818 (10.7), 163.0395 (2.9), 149.0230 (2.8), 136.9867 (2.7), 109.9994 (5.9) | 9.68 | 1.029 | 1,2,3,4,5 |
| **102** | Quercetagetin-3,6,3'(4')-trimethyl ether | $C_{18}H_{16}O_8$ | 359.0772 | 359.0777 (100), 344.0540 (76.5), 329.0306 (53.8), 314.0085 (2.7), 301.0356 (13.7), 286.0120 (21.9), 258.071 (17.8), 230.0217 (9.5), 214.0270 (2.5), 165.9903 (2.5), 163.0391 (0.7), 148.0162 (2.2), 136.9873 (0.5), 109.9999 (2.2) | 9.77 | 1.279 | 1,2,3,4,5 |
| **103** | Cirsimaritin (6-hydroxyapigenin-6,7-dimethyl ether) | $C_{17}H_{14}O_6$ | 313.0719 | 313.0721 (100), 298.0484 (53.2), 283.0249 (50.1), 269.0457 (3.5), 255.0295 (11.5), 227.0348 (4.2), 163.0026 (13.6), 135.0073 (4.0), 117.0332 (9.9) | 10.39 | 1.082 | 2,3,4,5,8 |
| **104** | Eupatilin/santin | $C_{18}H_{16}O_7$ | 343.0812 | 343.0825 (81.6), 328.0590 (100), 313.0357 (59.0), 298.0122 (11.8), 285.0407 (2.9), 270.0171 (43.7), 257.0095 (3.2), 242.0216 (3.7), 214.0265 (4.7), 198.0306 (0.7), 186.0314 (7.6), 164.9819 (1.8), 136.9866 (1.1), 133.0281 (3.1), 123.0437 (3.3) | 11.04 | | 1,2,3,4,5,6,9 |

| | | | | | | | |
|---|---|---|---|---|---|---|---|
| **105** | Casticin quercetagetin-tetramethyl ether | $C_{19}H_{18}O_8$ | 373.0932 | 373.0932 (100), 358.0696 (75.5), 343.0461 (71.5), 328.0232 (4.8), 315.0508 (2.1), 312.9998 (6.3), 300.0279 (13.1), 285.0042 (32.5), 257.0091 (20.0), 241.0140 (3.6), 229.0140 (7.9), 213.0185 (4.0), 185.0233 (1.0), 164.9821 (0.8), 163.0389 (0.8), 148.0158 (0.9) | 11.33 | 0.347 | 1,2,3,4,5 |
| | **Tentatively annotated compound** | **Molecular formula** | **Exact mass [M+H]$^+$** | **Fragmentation pattern in (+) ESI-MS/MS** | **$t_R$ (min)** | **Δ ppm** | **Distribution** |
| | | | | **Sesquiterpene lactones and derivatives** | | | |
| **106** | Dehydrocostus lactone[a] | $C_{15}H_{18}O_2$ | 231.1376 | 231.1375 (74.8), 213.1270 (19.6), 203.1432 (19.6), 195.1166 (10.6), 189.0906 (5.9), 185.1322 (100), 175.0751 (21.2), 159.1166 (29.2), 143.0853 (26.5), 131.0854 (30.9), 119.0856 (8.3), 105.0701 (29.6), 103.0545 (3.4), 95.0859 (26.1), 93.0703 (8.1), 91.0546 (14.2), 81.0704 (11.2), 67.0549 (4.4), 55.0551 (3.6) | 8.51 | -1.672 | 1,2,3,4,5,6,7,8,9 |
| **107** | Hydroxyalantolactone | $C_{15}H_{20}O_3$ | 249.1480 | 249.1479 (46.9), 231.1375 (26.7), 213.1268 (11.9), 203.1426 (7.5), 185.1321 (16.7), 175.1470 (0.8), 177.1270 (21.7), 159.1167 (5.9), 143.0853 (5.5), 131.0854 (5.9), 119.0856 (7.9), 105.0701 (10.9), 97.0651 (100), 91.0546 (5.7), 79.0548 (5.8), 67.0549 (3.3), 55.0186 (1.9) | 9.04 | -1.971 | 1,2,3,4,5,6,7,8,9 |
| **108** | Isoalantolactone[a] | $C_{15}H_{20}O_2$ | 233.1533 | 233.1532 (81.1), 215.1427 (55.6), 205.1584 (51.9), 197.1323 (21.9), 187.1478 (100), 161.1322 (20.3), 159.1166 (22.8), 147.1166 (48.6), 145.1166 (37.9), 133.1011 (23.1), 131.0855 (40.4), 119.0856 (32.1), 117.0700 (13.7), 105.0702 (55.4), 95.0859 (30.1), 91.0547 (28.9), 81.0704 (26.9), 79.0548 (14.2), 67.0549 (12.9), 55.0550 (8.8) | 9.74 | -1.357 | 1,2,3,4,5,6,7,8,9 |
| **109** | Partenolide[a] | $C_{15}H_{20}O_3$ | 249.1482 | 249.1476 (40.5), 231.1376 (100), 213.1268 (73.4), 203.1427 (41.7), 195.1165 (19.5), 185.1322 (81.4), 173.0960 (30.2), 145.1003 (72.9), 131.0857 (46.3), 119.0856 (28.7), 105.0703 (47.0), 91.0546 (29.3), 81.0704 (17.9), 67.0550 (8.9), 55.0550 (3.3) | 10.09 | -1.329 | 1,2,3,4,5,6,7,8,9 |
| **110** | Dehydroalantolactone | $C_{15}H_{18}O_2$ | 231.1376 | 231.1376 (99.5), 213.1271 (29.3), 203.1432 (6.6), 185.1323 (100), 175.0752 (17.3), 157.1010 (24.2), 143.0854 (30.8), 131.0855 (36.9), 119.0855 (12.7), 105.0702 (29.9), 95.0859 (54.1), 91.0546 (21.9), 81.0704 (20.6), 67.0550 (10.7), 55.0550 (6.8) | 10.62 | -1.412 | 1,2,3,4,5,6,7,8,9 |

| | | | | | | | |
|---|---|---|---|---|---|---|---|
| **111** | Curcumene | $C_{15}H_{22}$ | 203.1792 | 203.1792 (100), 175.1481 (5.6), 161.1324 (18.9), 159.1165 (0.7), 147.1166 (59.6), 135.1167 (5.9), 133.1011 (27.7), 121.1012 (14.1), 119.0856 (31.1), 109.1014 (49.7), 95.0859 (49.7), 93.0704 (12.2), 81.0704 (29.9), 79.0549 (6.7), 67.0549 (13.4), 55.0551 (6.9) | 11.38 | -0.922 | 1,2,3,4,5,6,7,8,9 |
| **112** | Curcumene isomer | $C_{15}H_{22}$ | 203.1792 | 203.1790 (100), 175.1477 (5.21), 161.1321 (8.35), 133.1010 (12.99), 121.1012 (13.70), 107.0857 (27.57), 95.0859 (40.15), 93.0703 (12.33), 81.0704 (18.96), 67.0549 (9.90), 55.0550 (4.07) | 11.69 | -1.069 | 1,2,3,4,5,6,7,8,9 |
| **113** | Alantolactone[a] | $C_{15}H_{20}O_2$ | 233.1533 | 233.1530 (59.5), 215.1426 (60.4), 205.1583 (3.8), 197.1321 (19.7), 187.1478 (100), 159.1165 (30.4), 147.1165 (27.5), 133.1010 (27.3), 119.0856 (26.7), 117.0700 (12.6), 161.1322 (38.2), 145.1009 (38.8), 131.0854 (46.3), 117.0700 (12.6), 105.0701 (90.1), 95.0859 (36.2), 91.0546 (40.1), 81.0704 (39.9), 79.0548 (20.5), 67.0549 (14.2), 55.0550 (11.0) | 15.74 | -1.443 | 1,2,3,4,5,6,7,8,9 |
| **114** | Farnesene | $C_{15}H_{24}$ | 205.1948 | 205.1947 (54.0), 163.1475 (8.6), 149.1323 (68.5), 135.1166 (39.1), 121.1012 (100), 107.0857 (44.9), 97.1014 (2.8), 95.0859 (49.7), 81.0704 (58.9), 71.0862 (2.5), 69.0705 (27.1), 55.0551 (16.7) | 18.82 | -1.449 | 1,2,3,4,5,6,7,8,9 |

[a] compare to reference standard; 1-n-hexane extract; 2-ethyl acetate extract; 3-DCM extract; 4-ethanol extract; 5-70% ethanol extract; 6-water extract; 7-n-hexane/β-CD; 8-ethyl acetate/β-CD; 9-DCM/β-CD.

**Table S2.** Secondary metabolites in *Inula sarana* extracts assayed by UHPLC-ESI-MS/MS in positive ion mode.

| No. | Identified/tentatively annotated compound | Molecular formula | Exact mass $[M+H]^+$ | Fragmentation pattern in (+) ESI-MS/MS | Δppm |
|---|---|---|---|---|---|
| | | | **Hydroxybenzoic, hydroxycinnamic and acylquinic acids, and derivatives** | | |
| **5** | Protocatechuic acid[a] | $C_7H_6O_4$ | 155.0338 | 155.0339 (15.5), 137.0232 (20.1), 111.0443 (100), 93.339 (57.0), 65.0393 (53.3) | 0.095 |
| **7** | Neochlorogenic (3-caffeoylquinic) acid[a] | $C_{16}H_{18}O_9$ | 355.1023 | 163.0387 (100), 145.0285 (7.6), 135.0439 (14.9), 117.0337 (9.6), 89.0390 (5.7) | - |
| **12** | Vanillic acid[a] | $C_8H_8O_4$ | 169.0495 | 169.0482 (18.7), 151.0388 (24.2), 125.0597 (60.4), 111.0443 (100), 93.0339 (78.6), 65.0393 (69.9) | -1.747 |
| **24** | Caffeic acid[a] | $C_9H_8O_4$ | 181.0495 | 181.0494 (2.2), 163.0387 (100), 145.0283 (14.7), 135.0440 (21.6), 117.0336 (15.9), 89.0391 (15.4) | -0.858 |
| **15** | 4-Hydroxybenzoic acid | $C_7H_6O_3$ | 139.0389 | 139.0387 (10.4), 111.0442 (100), 93.0339 (59.7), 65.0393 (62.6) | -1.874 |
| **16** | 3-Hydroxybenzoic acid | $C_7H_6O_3$ | 139.0389 | 139.0389 (13.1), 121.0285 (6.4), 111.0444 (57.6), 95.0495 (100), 93.0340 (4.0), 65.0393 (9.6) | -0.580 |
| **21** | Chlorogenic (5-caffeoylquinic) acid[a] | $C_{16}H_{18}O_9$ | 355.1023 | 163.0387 (100), 145.0282 (9.0), 135.0439 (12.7), 117.0336 (8.1), 89.0391 (6.7) | - |
| **23** | 4-Caffeoylquinic acid[a] | $C_{16}H_{18}O_9$ | 355.1023 | 163.0387 (100), 145.0286 (7.7), 135.0438 (13.5), 117.0336 (7.0), 89.0392 (6.3) | - |
| **27** | p-Hydroxyphenylacetic acid | $C_8H_8O_3$ | 153.0546 | 153.0547 (2.3), 135.0439 (41.1), 107.0494 (100), 95.0495 (6.5) | 0.518 |
| **38** | 3,4-Dicaffeoylquinic acid[a] | $C_{25}H_{24}O_{12}$ | 517.1340 | 163.0387 (100), 145.0284 (10.1), 135.0439 (18.2), 117.0336 (10.7), 89.0389 (9.0) | - |
| **40** | 3,5-Dicaffeoylquinic acid[a] | $C_{25}H_{24}O_{12}$ | 517.1340 | 163.0387 (100), 145.0283 (11.1), 135.0439 (17.7), 117.0337 (11.6), 89.0391 (9.4) | - |
| **41** | 4,5-Dicaffeoylquinic acid | $C_{25}H_{24}O_{12}$ | 517.1340 | 163.0387 (100), 145.0282 (11.4), 135.0440 (17.5), 117.0336 (11.6), 89.0389 (6.8) | - |
| | | | **Flavonoids** | | |
| **68** | Quercetin 7-*O*-rutinoside | $C_{27}H_{30}O_{16}$ | 611.1606 | 611.1591 (17.4), 465.1018 (16.8), 303.0481 (100), 285.0391 (2.9), 257.0434 (1.1) | -2.522 |
| **69** | Rutin[a] | $C_{27}H_{30}O_{16}$ | 611.1606 | 611.1583 (1.5), 465.1015 (11.6), 303.0492 (100), 257.0427 (2.2), 229.0492 (3.0), 165.0180 (1.5), 153.0178 (2.8), 137.0230 (2.8) | -3.863 |
| **71** | Isoquercitrin | $C_{21}H_{20}O_{12}$ | 465.1028 | 465.1022 (2.5), 303.0495 (100), 285.0380 (0.6), 274.0459 (0.5), 257.0429 (1.9), 229.0499 (3.5), 165.0180 (2.0), 137.0236 (4.0), 153.0183 (3.9), | 0.059 |
| **72** | Luteolin 7-*O*-neohesperidoside/rutinoside | $C_{27}H_{30}O_{15}$ | 595.1657 | 595.1639 (17.4), 449.1064 (29.0), 287.0542 (100), 153.0179 (4.4), 135.0439 (1.4) | -3.136 |

| | | | | | |
|---|---|---|---|---|---|
| **73** | Patuletin *O*-rutinoside | $C_{28}H_{32}O_{17}$ | 641.1712 | 641.1681 (1.1), 486.1123 (13.2), 333.0595 (100), 318.0362 (22.7), 273.0385 (1.6), 261.0360 (0.3), 244.0366 (1.9), 169.0126 (0.5), 137.0231 (1.1), | 4.891 |
| **74** | Hyperoside | $C_{21}H_{20}O_{12}$ | 465.1028 | 465.1028 (5.6), 303.0495 (100), 285.0379 (0.5), 274.0461 (0.3), 257.0439 (1.5), 229.0490 (2.8), 165.0182 (1.4), 137.0235 (2.6), 153.0182 (8.0), | -1.123 |
| **75** | Luteolin 7-*O*-glucoside[a] | $C_{21}H_{20}O_{11}$ | 449.1078 | 449.1066 (10.9), 287.0543 (100), 269.0439 (2.3), 241.0498 (0.3), 153.0181 (0.9), 121.0284 (1.1) | -2.756 |
| **76** | Patuletin *O*-hexoside | $C_{22}H_{22}O_{13}$ | 495.1133 | 495.1104 (1.5), 333.0595 (100), 318.0362 (18.6), 290.0414 (1.90, 273.0380 (1.9), 261.0396 (0.6), 244.0359 (2.2), 169.0127 (0.9), 137.0230 (2.0) | -5.811 |
| **78** | Kaempferol 3-*O*-rutinoside[a] | $C_{27}H_{30}O_{15}$ | 595.1657 | 595.1697 (1.6), 449.1062 (11.6), 287.0542 (100), 241.0506 (0.2), 231.0646 (0.4), 213.0541 (1.3), 165.0174 (1.8), 153.0182 (3.1), 121.0285 (2.1) | -3.863 |
| **79** | Nepetin *O*-hexoside | $C_{22}H_{22}O_{12}$ | 479.1184 | 479.1164 (14.6), 317.0650 (100), 302.0415 (43.6), 135.0444 (0.5), 137.0235 (2.2) | 4.200 |
| **80** | Nepetin *O*-rutinoside | $C_{28}H_{32}O_{16}$ | 625.1763 | 625.1724 (0.9), 479.1175 (13.1), 317.0649 (100), 302.0414 (23.9), 274.0450 (1.5), 256.0363 (0.4), 229.0490 (1.1), 147.0650 (1.4), 129.0545 (3.6) | -6.320 |
| **81** | Kaempferol 3-*O*-glucoside[a] | $C_{21}H_{20}O_{11}$ | 449.1078 | 449.1052 (25.3), 287.0544 (100), 153.0182 (2.1) | -5.896 |
| **82** | Isorhamnetin 3-*O*-glucoside[a,b] | $C_{22}H_{22}O_{12}$ | 479.1184 | 479.1188 (1.4), 317.0649 (100), 302.0415 (6.5), 274.0463 (3.9), 246.0512 (1.8), 229.0493 (3.3), 165.0182 (0.3), 153.0181 (7.4) | -0.005 |
| **83** | Chrysoeriol *O*-hexoside | $C_{22}H_{22}O_{11}$ | 463.1234 | 463.1225 (18.1), 301.0699 (100), 286.0465 (34.7), 258.0518 (11.4), 229.0491 (0.3), 168.0050 (3.3) | -2.133 |
| **84** | Cirsiliol *O*-hexoside | $C_{23}H_{24}O_{12}$ | 493.1340 | 493.1330 (16.9), 331.0802 (100), 315.0491 (10.4), 301.0326 (1.8), 298.0462 (5.4), 273.0387 (12.0) | |
| **85** | Spinacetin | $C_{17}H_{14}O_8$ | 347.0761 | 347.0755 (100), 332.0518 (31.6), 317.0289 (4.8), 289.0338 (7.6), 261.0391 (5.4), 233.0438 (0.5), 168.0051 (8.4) | -1.942 |
| **86** | Luteolin[a] | $C_{15}H_{10}O_6$ | 287.0550 | 287.0544 (100), 269.0435 (0.8), 241.0490 (0.9), 231.0660 (0.1), 213.0550 (0.3), 203.0340 (0.2), 153.0182 (10.5), 137.0234 (1.5), 135.0441 (3.6), | -2.106 |
| **87** | Quercetin[a] | $C_{15}H_{10}O_7$ | 303.0499 | 303.0492 (100), 285.1217 (5.5), 257.0446 (1.7), 229.0493 (8.2), 153.0183 (7.9), 137.0233 (5.9) | -2.538 |
| **88** | Patuletin (6-methoxyquercetin) | $C_{16}H_{12}O_8$ | 333.0605 | 333.0597 (100), 318.0364 (28.1), 273.0381 (2.7), 168.0051 (0.7), 137.0235 (3.3), | -2.413 |
| **89** | Nepetin (6-methoxyluteolin) | $C_{16}H_{12}O_7$ | 317.0656 | 317.0651 (100), 302.0418 (17.5), 274.0467 (30.8), 203.0336 (0.8), 153.0180 (0.9), 178.0869 (2.9), 132.0809 (3.5) | -1.637 |
| **90** | Axillarin | $C_{17}H_{14}O_8$ | 347.0761 | 347.0738 (100), 332.0518 (13.5), 317.0293 (2.7), 289.0336 (11.7), 168.0052 (1.6), 137.0233 (1.2) | -0.205 |
| **91** | Cirsiliol | $C_{17}H_{14}O_7$ | 331.0803 | 331.0806 (100), 315.0493 31.10), 301.0337 (1.1), 273.0389 (1.6), 245.0435 (0.1), 163.0179 (1.1), 137.0218 (0.1), 136.0147 (0.2) | -1.901 |
| **93** | Apigenin[a] | $C_{15}H_{10}O_5$ | 271.0601 | 271.0597 (100), 243.0635 (0.5), 229.0477 (0.3), 163.0383 (0.7), 153.0182 (10.1), 121.0285 (1.4), 119.0493 (5.5) | 8.61 |

| | | | | | |
|---|---|---|---|---|---|
| **94** | Quercetagetin 3,6,3'(4')-trimethyl ether | $C_{18}H_{16}O_8$ | 361.0918 | 361.0915 (100), 346.0676 (30.3), 331.0446 (1.4), 316.0229 (0.2), 257.0435 (2.1), 229.0493 (11.9), 168.0052 (7.0), 163.0385 (0.9), 139.0028 (0.3) | 1.112 |
| **96** | Hispidulin (scutellarein-6-methyl ether)[a] | $C_{16}H_{12}O_6$ | 301.0707 | 301.0700 (100), 286.0465 (24.0), 258.0517 (28.7), 229.0494 (1.9), 168.0050 (1.1), 121.0295 (0.4), | -0.372<br>-2.075 |
| **99** | Isorhamnetin[a] | $C_{16}H_{12}O_7$ | 317.0656 | 317.0650 (100), 302.0430 (4.2), 153.0182 (6.5) | -1.827 |
| **100** | Jaceosidin (6-hydroxyluteolin-6,3'-dimethyl ether)[a,b] | $C_{17}H_{14}O_7$ | 331.0812 | 331.0803 (100), 316.0570 (35.6), 301.0335 (7.4), 273.0388 (9.1), 245.0439 (6.7), 168.0051 (12.1) | -2.746 |
| **101** | Quercetagetin 3,6,3'(4')-trimethyl ether | $C_{18}H_{16}O_8$ | 361.0918 | 361.0909 (100), 346.0675 (24.3), 331.0426 (2.4), 316.0219 (0.3), 257.0440 (3.1), 229.0493 (18.3), 169.0131 (5.3), 137.0226 (0.5), 119.0493 (0.4) | -2.448 |
| **103** | Cirsimaritin (6-hydroxyapigenin-6,7-dimethyl ether)a,[b] | $C_{17}H_{14}O_6$ | 315.0863 | 315.0857 (100), 299.0546 (29.3), 285.0388 (1.4), 271.0597 (9.0), 242.0569 (2.4), 153.0182 (0.6) | -1.887 |

**Table S3.** The equations, range and $R^2$ values of standard compounds in the biological activity assays

| Assays | Equation | Range | $R^2$ |
|---|---|---|---|
| Total phenolic content (for Gallic acid) | Absorbance (y): 0.2199 x (µg gallic acid) | 0-5 µg | 0.9993 |
| Total flavonoid content (for Rutin) | Absorbance (y): 0.1274 x (µg rutin)+0.0506 | 0-20 µg | 0.9968 |
| | | | |
| DPPH radical scavenging (for Trolox) | Absorbance: 14.135x (µg trolox)+1.5632 | 0-5 µg | 0.9944 |
| ABTS radical scavenging (for Trolox) | Absorbance: 12.054x (µg trolox)-1.9894 | 0-2.5 µg | 0.9800 |
| CUPRAC (for Trolox) | Absorbance: 0.1328x (µg trolox)+0.002 | 0-2.5 µg | 0.9993 |
| FRAP (for Trolox) | Absorbance: 0.3166x (µg trolox)+0.003 | 0-2.5 µg | 0.9999 |
| Phosphomolybdenum (for Trolox) | Absorbance: 0.062x (µg trolox)-0.027 | 0-100 µg | 0.9998 |
| Metal chelating (for EDTA) | Absorbance: 13.314x (µg EDTA)-3.5881 | 0-4 µg | 0.9786 |
| | | | |
| AChE inhibition (for Galanthamine) | Absorbance: 185.44x (µg galanthamine)+0.5502 | 0-0.2 µg | 0.9962 |
| BChE inhibition (for Galanthamine) | Absorbance: 187.96x (µg galanthamine)-0.1896 | 0-0.2 µg | 0.9917 |
| Tyrosinase inhibition (for Kojic acid) | Absorbance: 1.5235x (mg kojic acid)+0.0055 | 0-0.3 mg | 0.9981 |
| Amylase inhibition (for Acarbose) | Absorbance: 2.0654x (µg acarbose)+4.0458 | 0-50 µg | 0.9543 |
| Glucosidase inhibition (for Acarbose) | Absorbance: 2.1183 (µg acarbose)-02336 | 0-50 µg | 0.9410 |

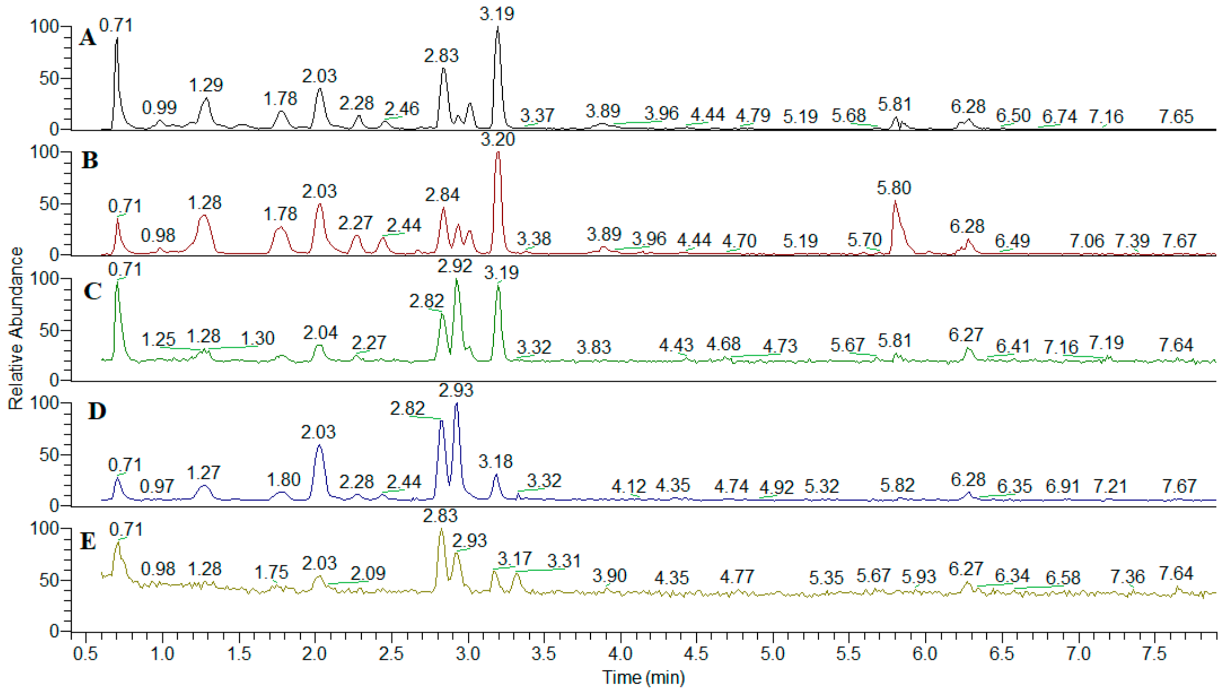


**Figure S1.** Extracted ion chromatograms of hydroxybenzoic and hydroxycinnamic acids and their derivatives; **A**—ethanol/water extract; **B**—ethanol extract; **C**—dichloromethane extract; **D**—ethyl acetate extract; **E**—hexane extract.

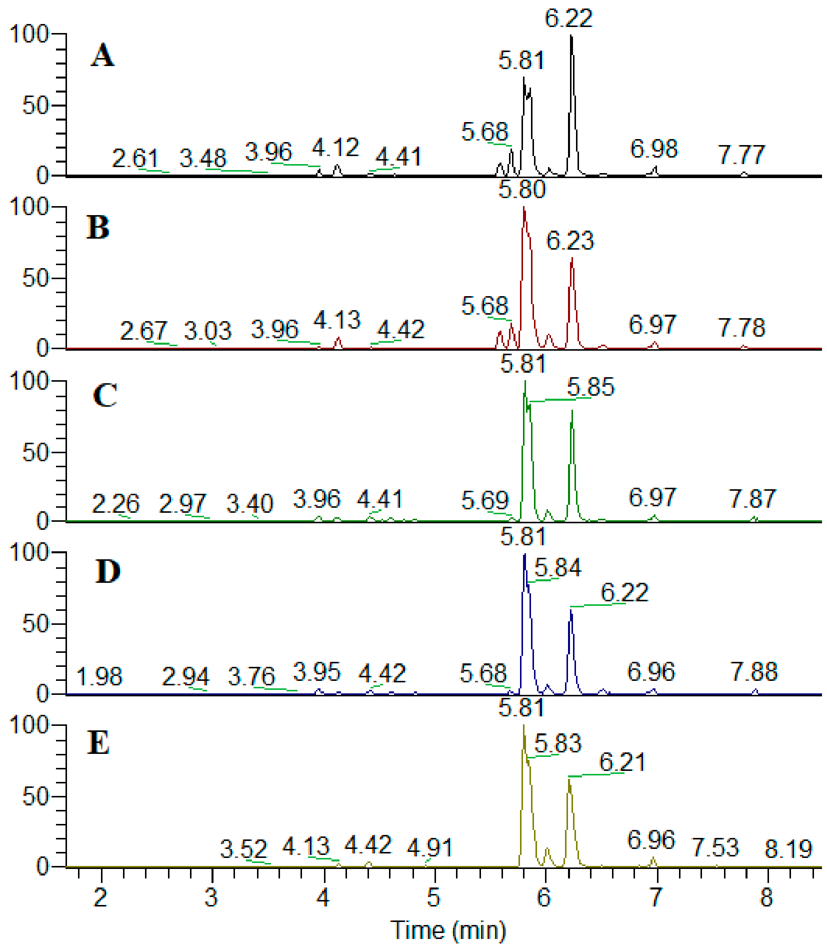


**Figure S2.** Extracted ion chromatograms of acylquinic acids; **A**—ethanol/water extract; **B**—ethanol extract; **C**—dichloromethane extract; **D**—ethyl acetate extract; **E**—hexane extract.

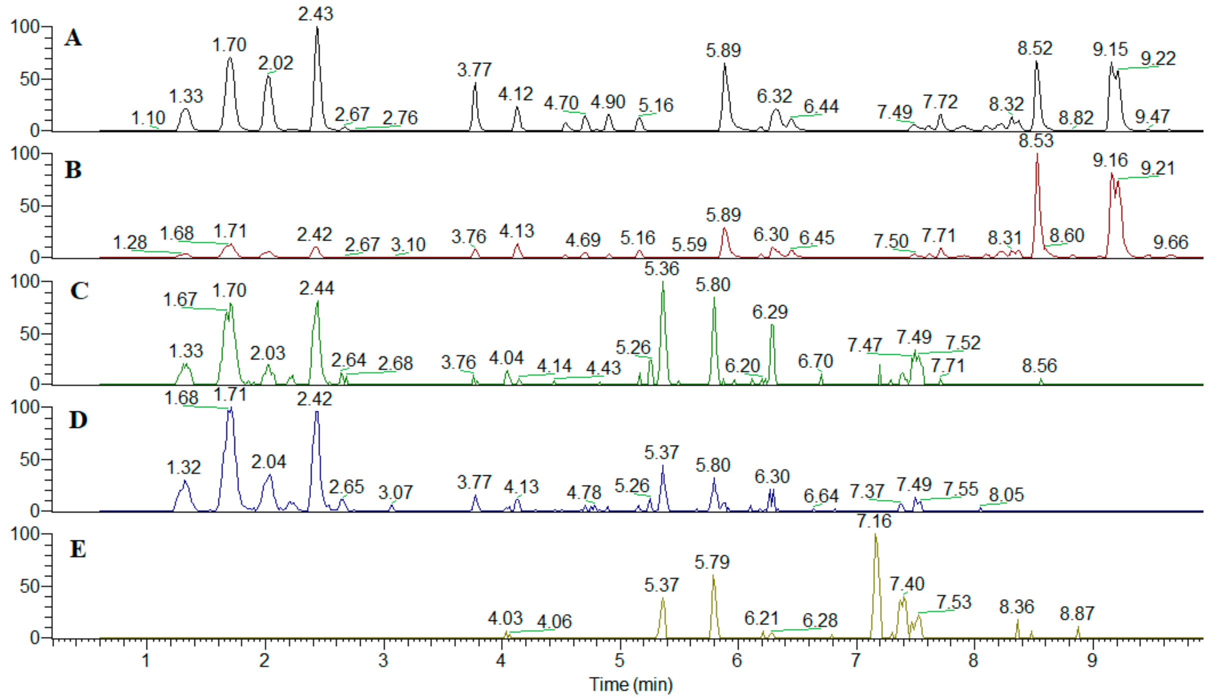


**Figure S3.** Extracted ion chromatograms of caffeoylhexaric acids; **A**—ethanol/water extract; **B**—ethanol extract; **C**—dichloromethane extract; **D**—ethyl acetate extract; **E**—hexane extract.

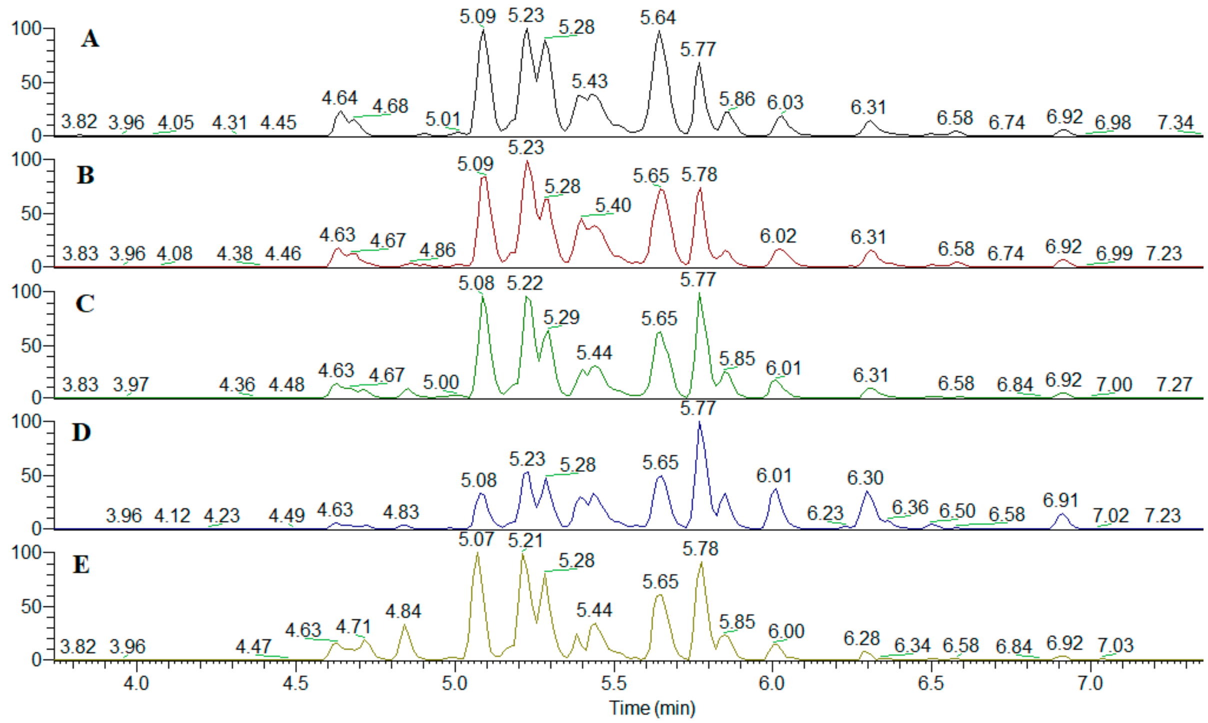


**Figure S4.** Extracted ion chromatograms of flavonoid glycosides; **A**—ethanol/water extract; **B**—ethanol extract; **C**—dichloromethane extract; **D**—ethyl acetate extract; **E**—hexane extract.

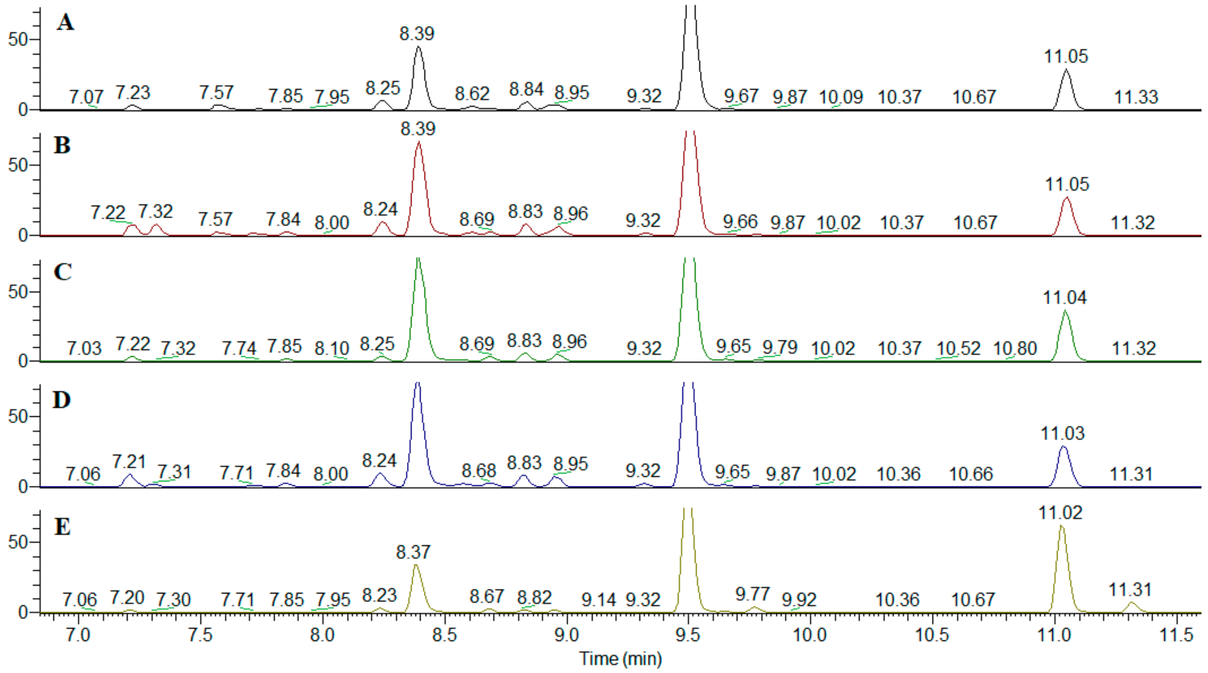


**Figure S5.** Extracted ion chromatograms of flavonoid aglycons; **A**—ethanol/water extract; **B**—ethanol extract; **C**—dichloromethane extract; **D**—ethyl acetate extract; **E**—hexane extract.

## References


Grochowski, D. M., Uysal, S., Aktumsek, A., Granica, S., Zengin, G., Ceylan, R., . . . Tomczyk, M. (2017). In vitro enzyme inhibitory properties, antioxidant activities, and phytochemical profile of Potentilla thuringiaca. *Phytochemistry Letters, 20*, 365-372.

Uysal, S., Zengin, G., Locatelli, M., Bahadori, M. B., Mocan, A., Bellagamba, G., . . . Aktumsek, A. (2017). Cytotoxic and enzyme inhibitory potential of two Potentilla species (P. speciosa L. and P. reptans Willd.) and their chemical composition. *Frontiers in pharmacology, 8*, 290.
